# Soft pinning: Experimental validation of static correlations in supercooled molecular glass-forming liquids

**DOI:** 10.1093/pnasnexus/pgad277

**Published:** 2023-08-25

**Authors:** Rajsekhar Das, Bhanu Prasad Bhowmik, Anand B Puthirath, Tharangattu N Narayanan, Smarajit Karmakar

**Affiliations:** Department of Chemistry, University of Texas at Austin, Austin, TX 78712, USA; TIFR Center for Interdisciplinary Science, Tata Institute of Fundamental Research, Hyderabad 500046, India; TIFR Center for Interdisciplinary Science, Tata Institute of Fundamental Research, Hyderabad 500046, India; Department of Chemical Physics, The Weizmann Institute of Science, Rehovot 76100, Israel; TIFR Center for Interdisciplinary Science, Tata Institute of Fundamental Research, Hyderabad 500046, India; Department of Materials Science and NanoEngineering, Rice University, 6100 Main Street, Houston, TX 77005, USA; TIFR Center for Interdisciplinary Science, Tata Institute of Fundamental Research, Hyderabad 500046, India; TIFR Center for Interdisciplinary Science, Tata Institute of Fundamental Research, Hyderabad 500046, India

**Keywords:** glass transition, amorphous order, molecular dynamics, supercooled Glycerol

## Abstract

Enormous enhancement in the viscosity of a liquid near its glass transition is a hallmark of glass transition. Within a class of theoretical frameworks, it is connected to growing many-body static correlations near the transition, often called “amorphous ordering.” At the same time, some theories do not invoke the existence of such a static length scale in the problem. Thus, proving the existence and possible estimation of the static length scales of amorphous order in different glass-forming liquids is very important to validate or falsify the predictions of these theories and unravel the true physics of glass formation. Experiments on molecular glass-forming liquids become pivotal in this scenario as the viscosity grows several folds ($∼10^{14}$), and simulations or colloidal glass experiments fail to access these required long-time scales. Here we design an experiment to extract the static length scales in molecular liquids using dilute amounts of another large molecule as a pinning site. Results from dielectric relaxation experiments on supercooled Glycerol with different pinning concentrations of Sorbitol and Glucose, as well as the simulations on a few model glass-forming liquids with pinning sites, indicate the versatility of the proposed method, opening possible new avenues to study the physics of glass transition in other molecular liquids.

**Significance Statement**

Unlike other phase transition, such as crystallization, glasses do not show any apparent order or dramatic structural changes when it develops rigidity. Nevertheless, various theories predict a hidden order, the “amorphous order,” which grows in the deep glassy regime. However, measuring such a static many-body correlation length in a glass former is difficult. Here, we propose a simple method, “pinning susceptibility,” which estimates the growth of amorphous order in glass-forming liquids within the random first-order transition theory. Combining computer simulations and dielectric spectroscopy experiments, we demonstrate that the proposed method captures the growth of amorphous order of the liquids and agrees with existing measurements by other methods.

## Introduction

One of the puzzling states of matter is undoubtedly the glassy state. Glasses are ubiquitous in nature, yet the physics of glass formation remained unsolved even after many decades of research efforts (1–4). Understanding the dynamical and mechanical properties of glass-forming liquids and solids is important as similar dynamical and mechanical behavior can be found in various complex systems, ranging from colloidal assemblies (5–8) to biological systems like cell membrane, cell migration, and bio-preservation (9–13). A detailed understanding of the fundamental properties of glass-forming systems will have far-reaching implications both in basic science and in industrial applications.

One of the major challenges in the field of glass transition is to understand the microscopic origin of the dramatic growth of viscosity of the glass-forming liquid upon a relatively small change in temperature while approaching the laboratory glass transition ($T_{G}$), defined empirically as the temperature where relaxation time reaches 100 s. Viscosity in some of these systems can change by 13–14 orders of magnitude upon ∼50–100 K change in temperature from its $T_{G}$. Another sought-after question is whether glass transition is a genuine thermodynamic phase transition or a purely dynamical crossover. There are a few existing theories that support both of these ideas. On the one hand, there is random first-order transition (RFOT) (14–16) theory, which predicts the glass transition to be a thermodynamic phase transition with growing static length, and the glassy state being an actual thermodynamic state. There are also frustration-limited domain theory (17–20) which provides another thermodynamic picture of glass transition. On the other hand, there is a class of theories that do not invoke the existence of a growing static correlation length to explain the growth of viscosity with supercooling. Kinetically constrained models (KCMs) (21) and mode-coupling theory (MCT) are such theories (22), which describe glass transition as a dynamic phenomenon associated with a diverging dynamic correlation length (22).

A completely different set of theoretical developments (23–25) assume that the local caging effect (local) along with the elastic effects coming from the neighboring particles (nonlocal) is the primary reason for the slowing down in the dynamics in deeply supercooled regime. In particular, in Refs. (26–28), the relaxation dynamics is pictured as an activated free energy barrier crossing process in which the barrier emerges from the caging effect, which is a noncooperative and local process. In contrast, another important contribution comes from the background collective effect due to elasticity. This theory is called elastically collective nonlinear Langevin equation (ECNLE) theory. This second process is nonlocal and cooperative. With supercooling, the contribution from both these processes leads to an increase in barrier height. However, the contribution from the elastic part increases much more rapidly than the caging contribution, and their relative dominance changes with changes in the fragility of the liquid (25, 29). Although this theory talks about cooperative behavior and activated dynamical processes associated with a free energy barrier, it does not directly associate a correlation length with the barrier height in contrast with the RFOT theory. Thus, it needs to be clarified whether a growing static correlation length is essential for understanding the rapid growth of viscosity as envisaged in RFOT theory (30). Similarly, whether one can associate an effective many-body correlation length even in ECNLE theory is an important question that needs further studies. Recent numerical studies on model systems that are inspired by such ideas of coupling between caging and effective elasticity of the medium are indeed very encouraging (31). Within these caveats, the subsequent part of our discussion will focus on estimating the elusive growing static correlation length as proposed in the thermodynamic theories of glass transition like RFOT.

Numerous works in the last few decades on the glass transition suggest the existence of multiple growing correlation lengths. A well-known growing length scale that can be identified is the dynamic heterogeneity length scale (32–35), which physically means the typical size of dynamically correlated regions in the system and are often quantified via four-point susceptibility, $\chi_{4}(t)$ (34, 36, 37) (see “Materials and methods” section for definition). On the other hand, the rapid growth of viscosity is often attributed to a growing static length scale (14, 38, 39). The static length scale is intrinsically related to structure and is commonly believed to refer to the growth of the so-called “amorphous order” (38, 40). One can consider the growth of amorphous order in supercooled liquid as the growth of a region where particles rearrange cooperatively. The region’s size is the measure of the static length scale. Estimating the static many-body correlation length is crucial to validate the predictions of these existing theories. Although measuring the correlation volumes in simulations is possible, it has its limitation of accessing the long-time scales relevant to experiments. On the other hand, measuring this correlation volume in experiments is extremely difficult, and there are only a few studies in the literature (41, 42). The difficulty mainly comes from the complicated nature of the correlation function that one needs to measure to estimate the length scale associated with the static correlations (41).

In Ref. (41), a fifth-order nonlinear dielectric susceptibility was measured to estimate the growth of the static correlation length. Note that a similar estimate of the growing static correlation done using third-order dielectric susceptibility in Ref. (43). This provides supporting evidence of a possible growing static correlation length in supercooled liquids approaching glass transition. It is also important to note in this context that the thermodynamic origin of glass transition as envisaged in RFOT theory with growing amorphous order and a static length scale is still actively debated, and there is a large body of evidence and theoretical arguments which suggest the transition can equally be a dynamic one without any growing static amorphous order as postulated in dynamic facilitation (DF) theory (21), MCT (22), ECNLE theory (25), etc. Recent experiments (42, 44) on dense colloidal systems near their glass transition density have pointed out that at lower densities, DF and MCT-like relaxation mechanisms play important roles in the dynamics, but at higher densities, more activated processes start to dominate the dynamical behavior which will be consistent with RFOT like mechanisms with a growing static length scale or ECNLE like mechanism without growth of any exotic static correlation length but with a subtle coupling between caging process and surrounding elastic processes control the dynamics. Thus, a proposal for new techniques which are experimentally accessible to measure the possible static length scale will surely be welcomed as it may provide critical inputs that are necessary to establish the correct mechanisms for glass formation and eventually lead to the development of the theory of glass transition.

In a recent work (45), it has been shown that it is possible to extract static length scale in a glass-forming liquid by measuring the system’s response in the presence of a small concentration of solute particles, which have smaller diffusion constants than the constituent molecules or particles of the supercooled liquid medium. The idea mainly originated from the previous works on the effects of random pinning (RP) sites on the dynamics of the supercooled liquids (46, 47). RP has recently been studied extensively to understand the possibility of ideal glass transition in these systems (46–51). As RP in a molecular liquid will be experimentally challenging, the idea of “soft” pinning sites was proposed (45). It was found that impurity particles with significantly smaller diffusion constants (more than an order of magnitude smaller than solvent particles (45)) behave like a pinning site for the particles of the liquid over a timescale comparable to the relaxation time of the liquid. Subsequently, a similar idea has been employed in colloidal glass experiments (44), in which certain particles which are immobile over a very large timescale compared to the bulk relaxation time ($\tau_{\alpha }$, see definition later) are chosen, and static correlation around that “soft pinning” (SP) sites have been studied to show that they pick up the same static correlation in the system. These results further strengthen that a solute particle with a significantly smaller diffusion constant indeed acts like a SP site. The effect of RP on relaxation dynamics can be understood within the ECNLE theory framework as well where pinning will essentially modify the caging and elastic coupling of the system leading to increase in relaxation time (52–55). The effect of pinning here is purely dynamical not connected to any structural changes. Thus extraction of a correlation length using pinning or SP is not necessarily proving the existence (or necessity) of such a length scale rather it proves that simulation and experimental results can be understood equally well within such a scenario.

**Fig. 1.**
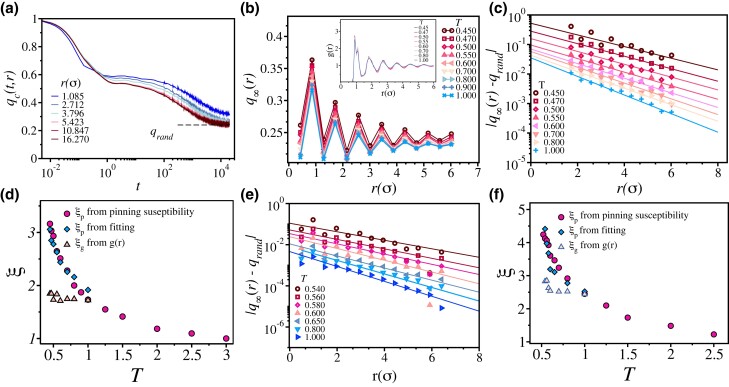
Estimation of length scales via RP. a) Time dependence of configurational overlap $q_{c}(t,r)$ at T=0.600 for the **2dR10** model. The dashed line correspond to the bulk value $q_{\mathrm{rand}}$. b) Variations of $q_{\infty }(r)$ as a function of *r* for **2dmKA** model. Inset shows the cross g(r) between the pinned particles and the mobile particles. In c) and e), $\left|q-q_{\mathrm{rand}}\right|$ is plotted as a function of *r* at all the studied temperatures for **2dmKA** and **2dR10** models, respectively. Data in (c) are multiplied by scaling factors for better clarity (see online supplementary material for details). Solid lines corresponds to exponential fits to obtain the correlation lengths. In d) and f), we compare the length scales estimated from the fitting with that obtained using pinning susceptibility (see text for details) for **2dmKA** and **2dR10** models, respectively. The value of $\xi_{p}$ estimated through two different methods matches well with each other. The length scales obtained using g(r) ($\xi_{g}$) are compared with other estimations. The growth of $\xi_{g}$ is much weaker than other lengths.

Assuming the existence of a static length scale $\xi_{s}$, a simple scaling theory can be developed if one assumes further that each pinning site slows down the relaxation process in its immediate neighborhood of linear size, $\xi_{s}$. Then the overall relaxation time of the entire system will be slower with increasing concentration (*c*) of the pinning sites. Now, if we assume that the relaxation processes around a pinning site is solely controlled by the underlying static length scale, then for small pinning concentrations, one would expect that the radial dependence of $\tau_{\alpha }$ from the pinning site will follow a scaling relation as


(1) $$\tau_{\alpha }(r,T)=\tau_{\alpha }(T)F[r/\xi_{s}(T)],$$

where F(x) is a scaling function which goes to 1 as x→∞. $\tau_{\alpha }(T)$ is obtained from the decay of the two-point density correlation function, Q(t) (defined in “Materials and methods” section) as $Q(\tau_{\alpha })=1/e$, where *e* is the base of the natural logarithm. The validity of the above scaling ansatz (Eq. 1) for the RP scenario has been presented in online supplementary material (see online supplementary Figs. S2 and S3, where we show $\tau_{\alpha }(r,T)$ rescaled by its r→∞ value as a function of *r* as well as the scaling collapse). If static length scale alone controls the relaxation dynamics, all the data should fall on top of each other if *r* (distance from the pinning cite) is rescaled by an appropriate length scale. The good quality of the data collapse using appropriate static correlation lengths suggests the approximation’s validity. The correlation length obtained from the decay of the peaks of two-point radial density correlation function g(r) from the pinning site does not grow as rapidly as the many-body correlation length of amorphous order (see Fig. 1d and f), which suggests that two-point structural length is not enough rather a many-body correlation length might be at play.

We can now approximately write the relaxation time for the entire system in the presence of *c* fraction of pinning sites (in the dilute limit) as


(2) $$\begin{array}{rl} \tau_{\alpha }(c,T) & ≃\frac{1}{N}\left[cN\rho \int_{0}^{\infty }[\tau_{\alpha }^{p}(r,T)-\tau_{\alpha }(T)]r^{2}\;dr+N\tau_{\alpha }(T)\right]\\ & =\tau_{\alpha }(T)\left[c\rho \int_{0}^{\infty }\left[F\left(\frac{r}{\xi_{s}(T)}\right)-1\right]r^{2}\;dr+1\right]\\ & =\tau_{\alpha }(T)\left[1+c\rho \xi_{s}^{3}\int_{0}^{\infty }[F(x)-1]x^{2}\;dx\right]\\ & =\tau_{\alpha }(T)(1+\kappa c\xi_{s}^{3}), \end{array}$$

where $\kappa =\rho \int_{0}^{\infty }[F(x)-1]x^{2}\;dx$ and ρ is the number density of the system. Thus, the scaling relation we arrive at is


(3) $$\frac{\tau_{\alpha }(c,T)}{\tau_{\alpha }(T)}≃1+\kappa c\xi_{s}^{3}\;\text{or}\;\log\;\left[\frac{\tau_{\alpha }(c,T)}{\tau_{\alpha }(T)}\right]≃\kappa c\xi_{s}^{3},$$

in the small pinning concentration limit. $c\xi_{s}^{3}$ remain much smaller than 1 for our experimental data, so we treat it as a small term in the expansion and then use log(1+x)≃x approximation to arrive at Eq. 3. Only when we combine existing literature data with ours, the scaling variable becomes closer to 1 and its continued validity at larger $c\xi_{s}^{3}$ is not immediately clear to us. Eq. 3 suggests that concentration dependence of the ratio of relaxation times is directly related to the underlying static length scale, which can be extracted via careful scaling analysis. Note that in Refs. (46, 47), detailed mathematical arguments based on RFTO theory also lead to the following scaling relation $\ln\;[\tau_{\alpha }(c,T)/\tau_{\alpha }(0,T)]=f[c\xi_{s}^{3}(T)]$, which is somewhat similar to Eq. 3 if the scaling function is Taylor expanded to keep the first two terms in the expansion in the small concentration limit. This argument suggests that if one can show that pinning sites do nucleate a correlated volume of size $\xi_{s}^{3}$, whose relaxation time is larger than the bulk, then the above-mentioned scaling ansatz will hold good, and the same can then be used to compute the underlying static length scale. Using Eq. 3, a new susceptibility, “pinning susceptibility” is introduced in Ref. (45) to estimate the static length directly, and it is defined as


(4) $$\chi_{p}(T,t)={\frac{\partial Q(T,c,t)}{\partial c}|}_{c=0},$$

where Q(T,c,t) is overlap function (see “Materials and methods” section). Using simple calculations and a few assumptions namely stretched exponential decay of Q(t) as $Q(t)∼\exp\;\left[-(t/\tau_{\alpha }{)}^{\beta }\right]$, where *β* is the stretching exponent) in Ref. (45), it was shown that $\chi_{p}^{\max}(T)∼\xi_{s}^{3}(T)$. The relation states that the peak value of $\chi_{\;p}(T)$ should directly measure the temperature dependence of $\xi_{s}$ upto an unknown scale factor. Using numerical simulation in Ref. (45), the validity of this relation has been confirmed. It has also been shown that this scaling theory works if one uses large particles with significantly lesser diffusion coefficients to act as “SP” sites. This finding makes this proposal very attractive for experiments, unlike hard pinning, as realizing SP in a natural system will be much easier. In the subsequent parts of this article, we employ some of these ideas and then extract the static length scale in both model glass-forming liquids via computer simulations and in supercooled Glycerol via dielectric experiments.

## Materials and methods

### Simulation details

We have performed extensive computer simulations of four well-studied model glass-forming liquids. They are referred in the text as **3dKA, 2dmKA, 3dR10**, and **2dR10** models, respectively. The first two models have active interatomic longer range interactions while the other two models are short range purely repulsive models. The choices of these models are to establish the generic nature of our results across models systems. The details of the model parameters are given in the online supplementary material.

We perform molecular dynamics simulations in a constant number of particles (*N*), volume (*V*), and temperature (*T*) (*NVT*) ensemble for the binary models. The integration was done using a modified leap-frog algorithm. To keep the temperature constant during the simulations at each studied temperatures, we use a Berendsen thermostat. Note that any other thermostat does not change the results qualitatively. Before storing the data, we equilibrate the systems well enough—at least run them for $100\tau_{\alpha }$. For the ternary model, we perform NPT molecular dynamics simulations using *LAMMPS* software (56). The pressure at each temperature is chosen to be the pressure of the corresponding binary mixture at that temperatures. For each studied temperatures, we have performed 32 independent simulations. For RP, we first equilibrate the system and then chose a fraction $c=\frac{N_{p}}{N}$ ($N_{p}$ is number of pinned particles ) of particles and fix them at their respective equilibrium positions. The rest of the particles are then evolved in time using normal Newtonian dynamics. For all the model systems, we use reduced units for the macroscopic variables. Lengths are measured in units of $\sigma_{AA}$, energy in units of $\epsilon_{AA}$ and time in units of $\sqrt{\frac{m\sigma_{AA}^{2}}{\epsilon_{AA}}}$ with m=1 being the mass of the particles.

### Correlation functions

#### Overlap correlation function

To characterize the dynamics, we calculated the two-point density–density correlation function or the overlap function defined as below (34):


(5) $$Q(T,c,t)=\frac{1}{N-Nc}\left[\left\langle \sum_{i=1}^{N-Nc}w(|{\vec{r}}_{i}(t)-{\vec{r}}_{i}(0)|)\right\rangle \right],$$

where the window function w(x)=1.0 if x≤0.30 and 0 otherwise and *N* is the total number of particles and *Nc* is number of pinned particles. ⟨…⟩ corresponds to the thermal averaging and […] corresponds to the averaging over different realizations of RP configurations. Any possible de-correlation of particles due to the rattling motion inside the cage formed by neighboring particles is removed by the window function. The relaxation time $\tau_{\alpha }$ is defined as $Q(t=\tau_{\alpha })=1/e$. For the pinned system, however we calculate the overlap function for only the mobile particles.

#### Four-point correlation function

The four-point susceptibility which estimates the degree of dynamic heterogeneity is defined as the fluctuations (34, 37, 39) in the overlap function and is given by, $\chi_{4}(t)=N[\langle Q^{2}(t)\rangle -\langle Q(t)\rangle^{2}]$. The four-point susceptibility defined above generally estimates the overall extent of dynamic heterogeneity of the system rounded by the finite system size.

### Experimental details

To calculate the dielectric constant, we used a parallel plate capacitor kept inside an aluminum cup which we call a sample holder. The electrodes of the capacitor are made of stainless steel to avoid any kind of chemical reaction with the samples. Teflon plates are used to avoid contact between electrodes and the sample holder. The separation between two electrodes is 1 mm, and the area of the electrodes is 36 cm${}^{2}$. The sample holder was connected to a solid massive Aluminum rod of very large heat capacity. This whole structure was kept inside a Dewar flask to keep it thermally isolated from the environment. The schematic of the setup is shown in online supplementary Fig. S4.

#### Dielectric spectroscopy experiments

We performed dielectric spectroscopy of ultra-pure Glycerol and Sorbitol purchased from Sigma and used as such. Glycerol is a strong glass former and exhibits glass transition at 193 K. We used Sorbitol and Glucose as solute particles. Sorbitol and Glucose molecules have linear sizes that are roughly double of a Glycerol molecule. Thus, it should have a diffusion coefficient significantly smaller than Glycerol and can act as SP particles in supercooled Glycerol medium. We prepare the sample by mixing the appropriate amount of Glycerol and Sorbitol (or Glucose) according to their atomic mass at room temperature. While making the sample, special care has been taken so that samples are homogeneously mixed and do not get any contamination. The obtained data is cross verified with the previously reported data from Ref. (57) (see online supplementary Figs. S11 and S12 for details). Details of data acquisition are given in online supplementary section V.

## Results

To understand the effect of particle pinning on the local dynamics in model glass-forming liquids, we follow the method developed in Ref. (58) and later used in Refs. (44, 59). The idea is to quantify the effect of pinned particles (small pinning fraction) on the local static order present in the system. To do so, we first study the overlap between configurations over time using configurational overlap $q_{c}(t)$ (44, 58, 59). For the estimation of $q_{c}(t)$, we first divide the simulation box into small grids of linear size 0.5σ, with *σ* being the particle diameter. Thus, one small box cannot accommodate more than one particle at any time. The configurational overlap $q_{c}(t)$ for each box is then defined as $q_{c}(t)=\frac{\left\langle n_{i}(t)n_{i}(0)\right\rangle }{\left\langle n_{i}(0)\right\rangle }$, where $n_{i}(t)=1$ if a particle occupies the box or else it is 0. We chose a pinned particle as center and then calculated the radially averaged value, $q_{c}(t,r)=\langle q_{c}(t)\rangle_{r}$ for all the boxes between *r* and r+dr. Particles close to the pinned particles will remain highly correlated to each other. However, as one goes further away from pinned particles, the effect will be diminished, and one will capture the bulk value. Thus, the decay of $q_{c}(t,r)$ will be slower for smaller $r^{\prime }s$. At long times, $q_{c}(t\to \infty ,r)$ reaches a plateau and oscillates around the bulk value $q_{\mathrm{rand}}$, the occupation probability of the individual box. Figure 1a shows a typical plot of $q_{c}(t,r)$ for various values of *r* for temperatures T=0.600 for **2dR10** model. Note that $q_{c}(t,r)$ reproduces the basic feature of the glassy liquids—the two-step relaxation which is prominent in Fig. 1a for T=0.600. Similar results for other temperature is shown in the online supplementary material.

### Random pinning

We first discuss the RP case before going to SP. We estimate the long-time value $q_{\infty }(r)$ of $q_{c}(t\to \infty ,r)$ by averaging the values at the plateau over time. In Fig. 1b, we show the oscillation of $q_{\infty }(r)$ around $q_{\mathrm{rand}}$ at various temperatures for **2dmKA** model. Similar results for **2dR10** model are shown in online supplementary Fig. S1c. The amplitude of the oscillations around $q_{\text{rand}}$ is higher close to the pinned particles, and it gradually decreases at longer distances. This behavior is similar to a pair correlation function, g(r), capturing the pinned particles’ density fluctuations. The envelope of each curve decays exponentially as a function of *r* from the pinned center. Therefore, the decay $\left|q-q_{\mathrm{rand}}\right|$ as a function of *r* will give the estimate of the static correlation length. In Fig. 1c, we depict $\left|q-q_{\mathrm{rand}}\right|$ as a function of *r* for **2dmKA** and Fig. 1e shows the results for **2dR10**. The solid lines are fits to the data using $\left|q-q_{\mathrm{rand}}|=A\exp\;(-r/\xi \right)$, where *A* and ξ are fitting parameters.

The length scale obtained from the fitting increases with decreasing temperatures (Fig. 1d and f). We want to emphasize here that the length scale obtained in this way matches well (see Fig. 1d and f for **2dmKA** and **2dR10**, respectively) with the pinning length scale obtained using RP scaling analysis described in Refs. (45, 46). Thus, the method “pinning susceptibility” (for details see Ref. (45)) derived within the the framework of RFOT captures the static length scale (in this case, the pinning length scale) of the system unambiguously. In the inset of Fig. 1b, we show g(r) computed from the pinning site and averaged over all the pinning particles in the system for different temperatures. The length scale, $\xi_{g}$, obtained from the decay of the peaks of g(r) is shown in Fig. 1d and f along with the amorphous order length scale. The variation of $\xi_{g}$ is much smaller than $\xi_{p}$, indicating the many-body nature of the amorphous order length scale. In the subsequent sections, we show how “pinning susceptibility” using an “SP” site can also be used in a similar manner to extract the static length scale of the system.

### Soft pinning

Recently (44, 60), the particle pinning method has been implemented elegantly in experiments of colloidal glasses. However, RP is still quite challenging to implement in many experiments, particularly in molecular glasses. As discussed in our earlier work (45) and shown in the subsequent paragraphs, the “SP” method is a possible way to realize a similar effect as that of RP. We devote the next part of our discussion to the effect of “SP” on the dynamics of glass-forming liquids both numerically and experimentally. To achieve the effect of particle pinning, we added a few impurity particles having a larger diameter than the parent liquid particles. According to the well-known Stokes–Einstein relation, particles with bigger diameters will have smaller diffusion constants. They will move much slower than the solvent particles (45) over their timescale of structural relaxation. Note that adding impurity particles at constant volume will increase the bulk pressure of the system, so we perform simulation keeping the pressure constant at that of the pure system at the particular temperature (see “Materials and methods” sections for details). Following the definition in Ref. (42), we identify the SP particles by first dividing the simulation box in a grid of linear size l=σ. Then we compute $q_{c}(t)$ for all the impurity particles and those having $q_{c}(t)>0.85$ for $t∼(5-10\tau_{\alpha })$ are chosen as “soft pinned” particles. Effectively those particles do not move much over the time scale $5\tau_{\alpha }$–$10\tau_{\alpha }$ and behave like randomly pinned particles. Once these soft pinned particles are identified, we repeat the similar analysis described in the earlier section (with l=0.5σ) to obtain the underlying length scale.

**Fig. 2.**
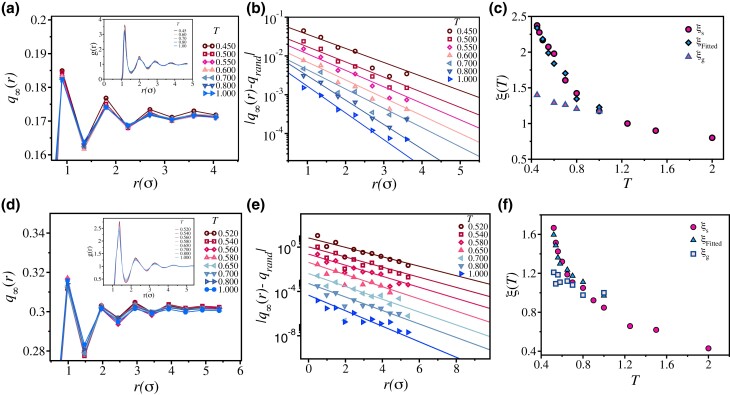
Estimation of length scales via SP. a) and d) $q_{\infty }(r)$ as a function of r(σ) for two different three-dimensional ternary model systems—a) **3dKA** and d) **3dR10** models, respectively. The insets show the corresponding cross g(r) between the soft pinned particles and the matrix. In b) and e), $\left|q-q_{\mathrm{rand}}\right|$ is plotted as a function of *r* at all the studied temperatures for **3dKA** and **3dR10** models, respectively. Solid lines correspond to exponential fits similar to as shown in Fig. 1. We then compare the estimated length scales with the one obtained previously using various methods (see text for details) in c) and f) for **3dKA** and **3dR10** models, respectively. Note that the static length scale estimated from the fitting (panels b and d) matches with $\xi_{s}$ (using various techniques). However, note that the growth of $\xi_{g}$ is much slower.

In Fig. 2a and d, we show $q_{\infty }(r)$ as a function of *r* for **3dKA** model and **3dR10** ternary models, respectively. Details of these models are given in “Materials and methods” section. Interestingly, note that the oscillation of $q_{\infty }(r)$ is similar to that observed in the case of RP. The oscillation amplitude is substantial for small *r*, and it dies out at larger *r*. Therefore, the effect of SP particles on the dynamics is similar but a bit weaker to that of actually pinned particles, and extends up to a distance *r* comparable to the static length scale of the system. Similar to the RP case, here also, the envelope of each curve ($q_{\infty }(r)$ vs *r*) decays exponentially. In Fig. 2b and e, we show the exponential decay of $\left|q_{\infty }-q_{\mathrm{rand}}\right|$ as a function of *r* for the **3dKA** and **3dR10** ternary models, respectively. The solid lines are the fits to exponential function to estimate the static length scales. The length estimated from the fitting is compared with the length scales obtained from other conventional methods like the point-to-set (PTS) method (see online supplementary material for details), Finite Size Scaling of α-relaxation time (for details see Ref. (45)) and Finite Size Scaling of Minimum Eigenvalue of Hessian Matrix (for details see Ref. (61)) in panels c and f of Fig. 2 for **3dKA** and **3dR10** models, respectively. The good agreement between the extracted length scales from these analyses and previously reported results demonstrates the reliability of the “SP” method in studying the growing static correlations in molecular liquids and prompt us to test the validity of this method in supercooled Glycerol using dielectric spectroscopy experiments, as discussed in the next section.

### Dielectric experiments—Glycerol–Sorbitol mixture

**Fig. 3.**
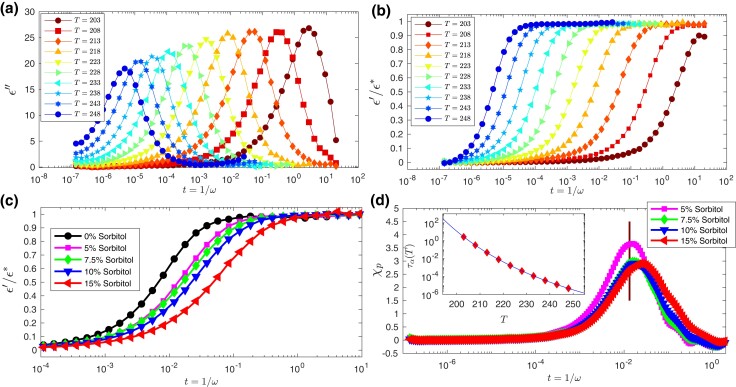
Pinning susceptibility in experiments. a) Time evolution of dielectric loss ($\epsilon^{\prime \prime }$) of pure Glycerol. The peak of $\epsilon^{\prime \prime }$ at any particular temperature gives the measure of α relaxation time at that temperature. Here we define time as inverse of the frequency as t=1/ω, with ω being the frequency. The time at which $\epsilon^{\prime \prime }$ has maximum value moves to larger time as temperature decreases which manifests the dynamic slowing down. b) Variation of dielectric constant ($\epsilon^{\prime }=\epsilon_{r}^{\prime }-\epsilon_{\infty }^{\prime }$) as a function of time (in seconds) for different temperatures for pure Glycerol. $\epsilon_{r}^{\prime }$ is the raw data from experiments and $\epsilon_{\infty }^{\prime }$ is the high frequency value of $\epsilon_{r}^{\prime }$. Note that $\epsilon^{\prime }$ is also rescaled between 0 and 1 using Havriliak–Negami fitting function (62) where $\epsilon^{*}=\epsilon_{s}-\epsilon_{\infty }$ and $\epsilon_{s}$ is the low frequency plateau value. c) Variation of $\epsilon^{\prime }$ at T=218 K for various Sorbitol concentrations. The data in the solid circle refers to pure Glycerol data. Data in solid square, solid diamond, down triangle, and left triangle refer to four different Sorbitol concentrations, 5.0, 7.5 10, and 15%, respectively. d) Pinning susceptibility, $\chi_{p}$ computed at T=218 K using these four different concentrations of Sorbitol in Glycerol. Vertical line indicates $1.6\tau_{\alpha }$ value of pure Glycerol. Inset shows the $\tau_{\alpha }$ as a function of temperature. The solid black line stands for Vogel-Fulcher-Tammann (VFT) fit to extract the Kauzmann temperature, which turns out to be $T_{VFT}≃122$ K in complete agreement with previously reported results (57).

We now come to the experimental verification of our “SP” method using dielectric spectroscopy measurements on supercooled Glycerol using Sorbitol as a “SP” co-solvent. We present only the salient features of our experimental results, and the details of the experimental procedures are described in “Materials and methods” section. In Fig. 3a and b, we show the variation of the dielectric loss ($\epsilon^{\prime \prime }$) and dielectric constant ($\epsilon^{\prime }$) of pure Glycerol as a function of time at different temperatures. Dielectric constant is rescaled by first subtracting the high frequency value, $\epsilon_{\infty }^{\prime }$ and then dividing by $\epsilon_{s}^{\prime }-\epsilon_{\infty }^{\prime }$, where $\epsilon_{s}^{\prime }$ is the low frequency plateau value. These constants for each temperature are obtained by fitting the data using Havriliak–Negami fitting function (62) and then rescaling makes it to vary between 0 and 1 (see online supplementary Figs. S7–S9 and section VII for details). The α-relaxation time ($\tau_{\alpha }$) of the system is obtained from the peak position of $\epsilon^{\prime \prime }$. The peak position shifts to the higher time with decreasing temperature, which clearly indicates slower dynamics at lower temperatures. The temperature dependency of $\tau_{\alpha }$ is shown in the inset of Fig. 3d. We fit these data using well known Vogel-Fulcher-Tammann (VFT) relation $\tau =\tau_{0}\exp\;\left(\frac{A}{T-T_{\mathrm{VFT}}}\right)$ and estimated the VFT temperature $T_{\mathrm{VFT}}≃122$ K and the calorimetric glass transition temperature of Glycerol, $T_{G}≃195$ K. $T_{G}$ is defined as the temperature at which the relaxation time becomes 100 s. These results are in close agreement with the value reported in (57). In Fig. 3c, we show the rescaled dielectric constant at T=218 K for various concentrations of Sorbitol in Glycerol. The data represented by black circle refers to pure Glycerol at T=218 K. Data in magenta (square), green (diamond), blue (left triangle), red (down triangle) refer to four different Sorbitol concentrations, 5.0, 7.5 10, and 15%, respectively.

We have defined the pinning susceptibility from the dependence of $\epsilon^{\prime }$ on the Sorbitol concentration (*c*) as


(6) $$\chi_{p}(t)=\partial \epsilon^{\prime }(t)/\partial c.$$

We use $\epsilon^{\prime }$ as a close proxy for the two-point density correlation function Q(t) as measured in the computer simulations. Any other suitable choice of the dynamical quantity can be used to define the pinning susceptibility without any loss of generality. Figure 3d presents the pinning susceptibility, $\chi_{p}$. The four datasets are for the Sorbitol concentrations of 5.0, 7.5, 10, and 15%, respectively. The time scale where $\chi_{p}$ exhibits a peak is proportional to relaxation time of the system. The vertical line in Fig. 3d, indicates the $1.6\tau_{\alpha }$ of the pure Glycerol. The peak height, which is supposed to measure the volume of the static correlation according to the scaling theory presented before, remains more or less constant with changing concentrations of Sorbitol. This result asserts that pinning susceptibility is a good measure of the growing correlations in molecular glass-forming liquids. The peak for 5% Sorbitol data is higher than the rest of the dataset, and we believe this stems from the measurement and subsequent analysis issues. First, $\epsilon^{\prime }$ needs to be normalized between 0 and 1 before $\chi_{p}$ can be calculated, and a small error in doing so can lead to variation in peak value. Also, note that $\chi_{p}$, computed from 5% Sorbitol concentration data, is systematically larger for all temperatures. Still, the temperature dependence is the same as other concentrations, as discussed in the subsequent paragraph. We want to briefly mention that although the relaxation time increases systematically with added Sorbitol, Q(t) changes in such a way that the peak value of susceptibility $\chi_{p}(T,t)$ do not change for small Sorbitol concentrations. If susceptibility were to change with *c*, we would obtain different length scales for each choice of *c*. Hence, for $\chi_{p}$ to accurately represent the correlation length, it should remain unchanged with variations in *c*, as long as *c* is sufficiently small. As a counterexample, if we select $\chi_{\rho }=\partial Q/\partial \rho$, where ρ is the density, as the susceptibility, it would consistently increase with increasing ρ (see Ref. (45)).

**Fig. 4.**
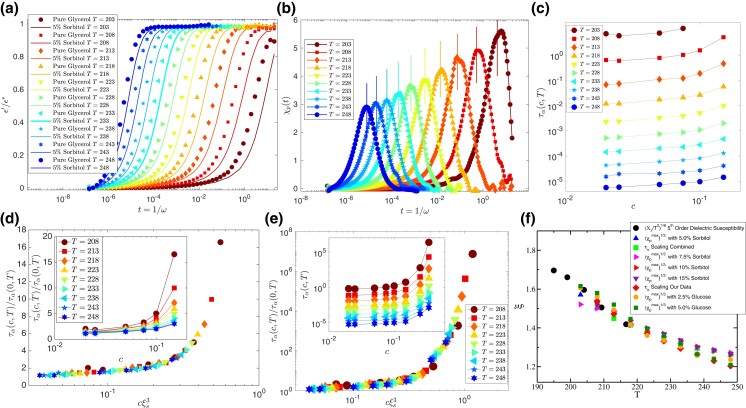
Estimation of static length scale in supercooled Glycerol. a) Time dependency of dielectric constant for pure Glycerol (symbols) and 5% Sorbitol mixture (solid lines). b) Pinning susceptibility as a function of time. The vertical lines indicates $1.6\tau_{\alpha }$ values for all the temperatures. This confirms that peak appears at timescale proportional to the typical relaxation time of the system. c) Variation of α relaxation time of Glycerol–Sorbitol mixture as a function of Sorbitol concentrations for different temperature. d) Data collapse of $\tau_{\alpha }$ using the scaling relation in Eq. 3. T=203 K data in not included in the scaling collapse (see text for details). e) Data collapse of α relaxation time of Glycerol–Sorbitol mixture. f) Comparison of static length scale found from our own experiment (shown as “$\tau_{\alpha }$ Scaling Our Data”) and calculated from the data provided in Ref. (63) (shown as “$\tau_{\alpha }$ Scaling Combined”) with the length scale data extracted from Ref. (41). Note that, the length scales estimated from the peak values of $\chi_{p}$ at various Sorbitol concentrations matches with each other. This surely is a benchmark supporting evidence of the validity of the scaling assumptions as well as the reliability of the obtained length scale.

In Fig. 4a, the variation of dielectric constant with time t=1/ω, where ω is the frequency, is shown for 5% concentration of Sorbitol (solid line) and pure Glycerol (filled symbols) in the temperature range 203 to 248 K. The unit of time is second. With increasing solute concentration, the relaxation becomes slower, which proves that the presence of larger Sorbitol molecules makes the dynamics slower. So these large particles can be thought of as “SP” sites for the Glycerol molecules. In Fig. 4b, the pinning susceptibility, calculated using Eq. 6, is shown. The maxima of $\chi_{p}(T,t)$ increases with decreasing temperature, manifesting the growth of amorphous order within the framework of RFOT theory. This is obtained for 5% Sorbitol concentration in Glycerol. The pinning susceptibility, $\chi_{p}$, obtained using c=7.5,10, and 15% (see online supplementary Fig. S10) yields the same value of the static correlation. The vertical lines in Fig. 4b indicate $1.6\tau_{\alpha }$ values of pure Glycerol at these temperatures.

Next, we concentrate our attention on the scaling analysis of relaxation time ($\tau_{\alpha }$) to test the correctness of the results via $\chi_{p}$ in a self-consistent manner. As we obtained relaxation time directly from the peak position of dielectric loss, $\epsilon^{\prime \prime }$ data, we can perform the scaling analysis without involving any fitting procedures. The relaxation time of Glycerol for various concentrations of Sorbitol is shown in Fig. 4c. Using the scaling relation, as shown in Eq. 3, we perform the scaling analysis presented in Fig. 4d. The inset of the same figure shows the data before scaling along the *x*-axis to extract the desired length scale. The data collapse is shown in Fig. 4d and observed to be very good, suggesting the excellent signal-to-noise ratio in experimental measurements. The data for T=203 K is not included in the data collapse as we do not have the relaxation time data for higher concentrations of Sorbitol, and the data is a bit noisy compared to other datasets. Although with incomplete data, one can still perform the scaling analysis without altering the results (see online supplementary Fig. S13 and section X for details). In Fig. 4f, the measured length scale is then compared with the existing values reported in Ref. (41) in which the fifth-order nonlinear dielectric susceptibility ($\chi_{5}(T,t)$) of Glycerol was measured and the length scale is given as $\xi_{s}(T)∼(\chi_{5}/T^{2}{)}^{{}^{\frac{1}{6}}}$. Again, the agreement is excellent for clearing away any doubt about the reliability of this innovative method. We want to emphasize that the growth of the static length scale in Glycerol in the studied temperature range is not large as Glycerol is a intermediate fragility glass former. Nevertheless, it explains the non-Arrhenius temperature dependence of $\tau_{\alpha }$ once we plot $\tau_{\alpha }$ as a function of $\xi_{s}/K_{B}T$ following the RFOT predictions (see “Relation between length and time scales” section for further details). Although it does not prove corretness of RFOT theory, it shows that results are consistent within that theoretical framework.

To re-validate the accuracy of our experimental results, we analyzed some of the previously published data. In Refs. (57, 63, 64), the α relaxation time of the Glycerol–Sorbitol mixture for various concentrations (mostly large concentrations) is reported. So, we have taken all the reported data for all the concentrations (see online supplementary Fig. S5 for the details of the data extraction and online supplementary Figs. S11 and S12 for consistency check of our data with the reported ones) and combined our data for smaller concentrations to perform the scaling analysis over the entire range of data available with us. In Fig. 4e, we show the scaling analysis of the entire dataset. Remarkably the data collapse is found to be very good for all the data of various concentrations. In Fig. 4f, we compare the length scale obtained from all the scaling analyses along with the static length that can be obtained from peak height of $\chi_{p}$ as $\xi_{s}∼\chi_{p}^{1/3}$ at various concentrations of Sorbitol. The comparison of the static length scales measured using all these various ways and the reported value from Ref. (41) are in good agreement with each other. Thus, our proposal of “soft pinning” to extract the static length scale in glass-forming molecular liquids is robust, and we hope that a careful analysis of experimental data can lead to understanding the growth of static length scales in various other molecular glass-forming liquids in the near future.

Although the scaling arguments (Eq. 3) are valid in the small concentration limit, they remain useful in describing the data at much larger concentrations without strong deviation. Similar results are obtained for model systems in simulations. At large concentrations of pinned particles, one can expect the percolation transition to dominate the behavior and see a qualitative change in the relaxation behavior at c>16%. The relaxation time data suggest that the effect is probably not very strong for variation of relaxation time with increasing concentration of (soft) pinning particles, as also seen from the simulation. In simulations, pinning concentration as high as 30% is used (51, 65). Previous studies indicate that the physics qualitatively changes once the pinning concentration is larger than 50%, as one will eventually cross over to Lorentz gas-like scenario (66, 67). However, it remains to be understood, why our results do not qualitatively change even when large concentration data are included in the analysis. We acknowlege that although we have taken utmost care during experiments, at high concentration of Sorbitol, possible clustering or nonrandom mixing effects cannot be ruled out.

### Dielectric analysis—Butanol–Hexanol mixture

**Fig. 5.**
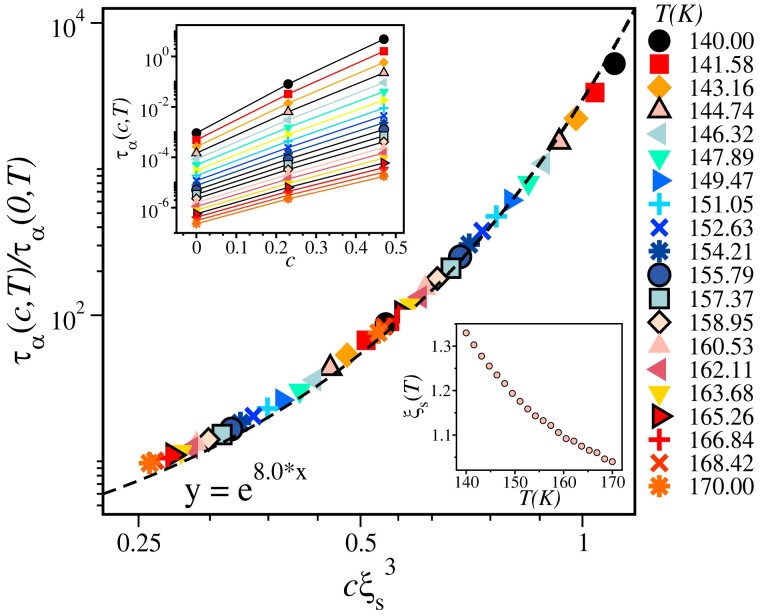
Static length scale in supercooled Butanol. Data collapse of $\tau_{\alpha }$ for Butanol–Hexanol mixture obtained using the scaling relation in Eq. 3 discussed in the text. The upper inset shows the extracted data ($\tau_{\alpha }$) of Butanol–Hexanol binary mixture as a function of different concentration of Hexanol. The lower inset shows the growth of the length scale as a function of temperature in glass-forming Butanol.

A similar analysis for Butanol using Hexanol as “SP” molecules is presented in Fig. 5. In the inset, we show the extracted data (see online supplementary section VI and Fig. S6) of the relaxation time of Butanol as a function of the concentration of the Hexanol. The data collapse obtained using the scaling ansatz Eq. 3 is again found to be good. The underlying growth of the length scale is shown in the inset of the same figure. Verification of these results using other scaling methods is not possible at this moment as we do not have the $\epsilon^{\prime }$ and $\epsilon^{\prime \prime }$ data available with us for this Butanol–Hexanol binary mixture with varying compositions. The fact that scaling analysis works so well even for this binary mixture of glass formers supports this method’s universality. The composition dependence dynamical quantities in binary glass-forming liquids are poorly understood. Often dependence of $T_{G}$ on composition is used to be fitted empirically via different functions; our scaling theory provides a unified description of the composition dependence of Glycerol–Sorbitol mixture over a large concentration regime. The beauty of the scaling collapse undoubtedly indicates an elegant way to understand the physics of a binary mixture of glass-forming liquids in experiments.

### Effect of interatomic interaction on pinning susceptibility

**Fig. 6.**
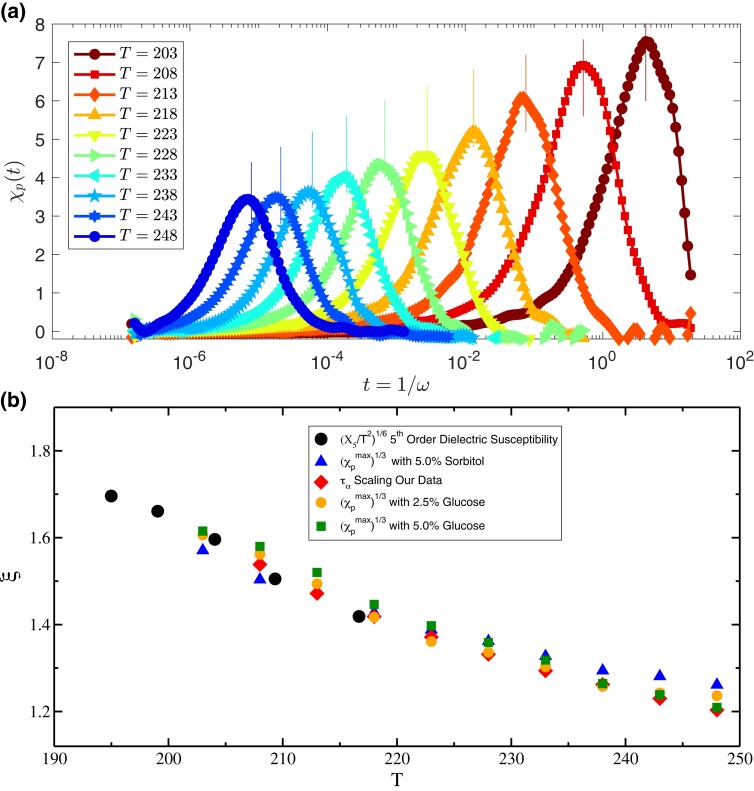
Dependence of pinning susceptibility on interatomic interactions. a) Pinning susceptibility, $\chi_{p}$ in supercooled Glycerol with Glucose being SP particles at a concentration of 2.5%. With decreasing temperature the peak height increases monotonically and also shifts to large time. The vertical lines indicate the $1.6\tau_{\alpha }$ values of the pure Glycerol. Note that the peak appears at the same time as that in the Sorbitol case. b) The temperature dependence of the correlation length obtained as $\xi_{s}\propto (\chi_{p}^{\max}{)}^{1/3}$ for Glucose concentration of 2.5 and 5%. The same estimate of the length scale using various concentrations of Sorbitol is also plotted for direct comparison. The near perfect agreement amongst these various estimates of the length scale, clearly establishes the robustness of the proposed method and its insensitivity on the details of the intermolecular interaction. See text for further discussion.

To understand the possible effect of interatomic interaction between the SP particles and the host liquid medium on the pinning susceptibility and the subsequent estimation of the correlation length, we changed the interaction potential (between the SP particles and the host liquid medium) in our simulation and recomputed the correlation length over a variety of parameter ranges. Our results clearly show (see online supplementary Fig. S14 for details) that the details of the interaction potential do not have any qualitative effect on the pinning susceptibility and the corresponding estimate of the temperature dependence of the underlying static correlation length. To reconfirm the same effect in our experiment on supercooled Glycerol, we performed another set of experiments with Glucose being the SP co-solute particles at two concentrations, 2.5 and 5%, respectively. In Fig. 6a, we show the pinning susceptibility for various temperatures at 2.5% Glucose concentration. One can see that the peak height of the pinning susceptibility, $\chi_{p}$, increases systematically, indicating the growth of the length scale. The vertical lines here also refer to the $1.6\tau_{\alpha }$ values of pure Glycerol at these temperatures. It is exciting to see that $\chi_{p}$ peaks appear at the same timescale irrespective of whether Sorbitol or Glucose are being used as SP molecules. In Fig. 6b, we show the obtained length scale $\xi \propto (\chi_{p}^{\max}{)}^{1/3}$ as a function of temperature for the two different concentrations. We also compare the length scale obtained using Sorbitol as the SP particle in the same figure. Excellent agreement amongst the different estimates of the correlation length establishes that pinning susceptibility is indeed a robust measure of static correlation length in molecular glass-forming liquids.

In Ref. (63), dynamical behavior of glass-forming liquids mixture is studied using KCM (68, 69). The changes in the relaxation times of liquid mixtures have been analyzed using arguments based on dynamic facilitation theory by computing an effective energy parameter with two adjustable parameters that lead to nice data collapse of a range of liquid mixtures. Thus, it is not immediately clear whether one can ascertain that an RFOT picture or dynamic facilitation mechanisms are in play in the glass transition, but it suggests that an alternate description might be possible to describe the relaxation behavior of glass-forming liquid mixtures. It is very tempting to hypothesize that the discrepancy observed in Glycerol–Sorbitol data analysis in Ref. (63) probably suggests that RP and RFOT scenario may be at play when one is considering a liquid mixture with a large difference between the effective molecular diameter of the two liquid species and, thereby, a large difference in their respective glass transition temperatures. Conclusions drawn in another recent work (70) in which glass transition of dimer–polymer mixture is studied extensively in a composition regime where polymers play the role of pinning site in the medium of dimers are in clear agreement with our results.

### Relation between length and time scales

The growth of $\tau_{\alpha }$ and the corresponding growth of $\xi_{s}$ depend crucially on the nature of the glass former. For example, in the case of fragile glass formers, the timescale grows much more rapidly compared to a strong glass former (see Ref. (71)). Within RFOT picture, $\tau_{\alpha }$ is related to $\xi_{s}$ as $\tau_{\alpha }=\tau_{0}\exp\;(\Delta \xi_{s}^{\psi }/K_{B}T)$, where $E_{a}=\Delta \xi_{s}^{\psi }$ is the typical free energy cost required to rearrange a region of linear size $\xi_{s}$ and *ψ* is the RFOT exponent. Δ is a positive quantity and would be a weak function of temperature and all the temperature dependence of the barrier in various glass-forming liquids should come from the growth of $\xi_{s}$ as envisaged in RFOT theory. Therefore, the corresponding growth of length scales will also depend on the fragility. It is important to note that, even for fragile liquids, the static length scale grows only by a factor of 5–6 particle diameter, as reported in many previous studies (48, 58, 72). As Glycerol and Butanol fall in the moderate fragility category, it is not very surprising that the estimated length scales do not grow strongly. It increases by a factor of ∼1.5 for Glycerol and Butanol in the studied temperature range.

**Fig. 7.**
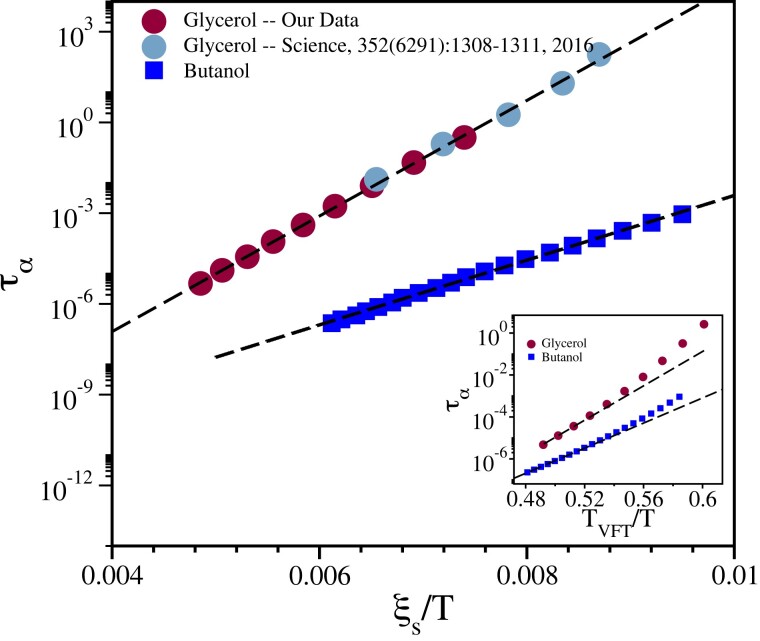
Relation between length and time scale. $\tau_{\alpha }$ is plotted as function $\xi_{s}/T$ with the straight line being the expected Arrhenius behavior for Glycerol (solid circles) and Butanol (solid squares). The inset shows that $\tau_{\alpha }$ vs 1/T cannot be fitted by a straight line. In the inset, we have plotted $\tau_{\alpha }$ as a function of $T_{\mathrm{VFT}}/T$ for better visualization.

To check whether the estimated length scales explain the super Arrhenius behavior of the studied liquids, we have plotted $\tau_{\alpha }$ as a function of $\xi_{s}/T$ in Fig. 7 for Glycerol and Butanol. Important to note that both Glycerol and Butanol follow the RFOT relation with ψ=1.0. We want to emphasize that $\tau_{\alpha }$ vs 1/T cannot be fitted by a straight line for both Glycerol and Butanol in the entire temperature regime (see insets of Fig. 7). So, small but finite growth of static correlation length is intimately related to the growth of relaxation time in these studied liquids. These results, although do not prove the validity of the RFOT-type picture of glass transition with a growing static length scale, they certainly establish its importance for understanding the puzzles of glass transition in the accessible temperature regimes. The future experimental measure of this correlation length in a wide variety of glass formers with varying fragility in the entire window of relaxation time covering 14 orders of magnitude may throw significant light on the ongoing debate on the validity of various theories that predict activated relaxation processes in the deep supercooled temperature regime with (15) and without (25) a growing static many-body correlation length.

## Discussions and conclusions

We performed extensive computer simulations of model glass-forming binary liquid mixtures with RP in two and three dimensions. We estimated the extent of correlation from the local pinning sites at each studied temperature to obtain the underlying static correlation length. The obtained correlation length is found to be in close agreement with the one obtained using other conventional methods like the PTS method. We then employed the idea of “SP” sites using impurity particles with larger diameters to see if they could act as pinning sites up to the structural relaxation time of the host liquid medium. As shown previously in colloidal experiments (44), we also found that larger particles can be excellent pinning sites, and correlation around them can be used to obtain the underlying correlation length. With this knowledge, we then developed a scaling theory to understand the dependence of the relaxation time of the host liquid medium on “SP” particles and measure the underlying static correlation length of the system. This scaling analysis rationalizes all the data obtained from our simulations in a unified manner. We then performed dielectric susceptibility experiments of Glycerol in the supercooled temperature regime with varying concentrations of Sorbitol and Glucose as SP particles. The correlation length extracted using this scaling analysis for Glycerol is then compared with the results obtained via measurement of fifth-order nonlinear dielectric susceptibility from Ref. (41). Excellent agreement between these two length scales establishes this simple method’s reliability and usefulness. We are very hopeful that by using this method, one will be able to obtain the correlation length and study its growth in various molecular liquids over a wide spread of fragility and test validity of theories of glass transition which predict growing static many-body correlation associated with growth of viscosity. Simulations and colloidal experiments are not a good substitute for this, as, in simulations and colloidal experiments, one can hardly access time scale change of $10^{3}$–$10^{4}$. So, even though simulations and colloidal experiments give more microscopic insight, they do so only within the high-temperature limit. To access the low-temperature dynamical properties of glass-forming liquids, molecular glass-forming liquids are ideal candidates. Thus, our proposed experimentally accessible method will certainly be beneficial in understanding the mechanisms of glass formation and developing a unified theory of glass formation, a problem that remained unsolved even after many decades of extensive research. Finally to reiterate, as the proposed scaling analysis and the estimation of the correlation length are done within the RFOT theory formalism assuming existence of growing static length scale, it does not prove the existence of an amorphous order length scale; instead, it shows that one can understand experimental and simulation results with the assumption of a growing static length as alternate theoretical framework (25) may equally be able to explain the data without directly invoking growth of such a static scale.

## Supplementary Material

pgad277_Supplementary_Data

## Data Availability

The data that support the findings of this study are available from the corresponding author upon reasonable request.

## Preprints

A preprint of this article is published at [arXiv:2106.06325].
