# Supplementary material for: Soft pinning: Experimental validation of static correlations in supercooled molecular glass-forming liquids
Source: PNAS Nexus. 2023 Aug 25;2(9):pgad277. doi: 10.1093/pnasnexus/pgad277 (PMC10482383; doi:10.1093/pnasnexus/pgad277)
Supplement: pgad277_Supplementary_Data [file pgad277_supplementary_data.pdf]

Soft-Pinning: Experimental Validation of Static Correlations in Supercooled Molecular Glass-forming Liquids –supplementary materials.

Rajsekhar Das[1,2], Bhanu Prasad Bhowmik[1,3], Anand B Puthirath[1,4], Tharangattu N. Narayanan[1], and Smarajit Karmakar[1*]
[1] *Centre for Interdisciplinary Sciences, Tata Institute of Fundamental Research, 36/P, Gopanpally Village, Serilingampally Mandal, Ranga Reddy District, Hyderabad, 500107, India*
[2] *University of Texas at Austin, USA*
[3] *Weizmann Institute of Science, Rehovot, Israel and*
[4] *Department of Materials Science and Nano Engineering, Rice University, 6100 Main Street, Houston, TX, 77005, USA*

I. MODEL DETAILS

We have performed extensive computer simulations of the following four well-studied model glass-forming liquids.
(i) 3dKA – The first model that we studied is the well-known Kob-Andersen model [1]. It is a 80 : 20 binary mixture of two type of particles with interacting potential

$$V_{\alpha\beta}(r) = 4\epsilon_{\alpha\beta}\left[\left(\frac{\sigma_{\alpha\beta}}{r}\right)^{12} - \left(\frac{\sigma_{\alpha\beta}}{r}\right)^{6}\right]. \tag{1}$$

The parameters of this model with $\alpha, \beta \in \{A, B\}$, are $\epsilon_{AA} = 1.0$, $\epsilon_{AB} = 1.5$, $\epsilon_{BB} = 0.5$, $\sigma_{AA} = 1.0$, $\sigma_{AB} = 0.80$, $\sigma_{BB} = 0.88$ and number density $\rho = 1.20$. The potential is cut off at a distance $r_{cut} = 2.50\sigma_{\alpha\beta}$. We use a quadratic polynomial in such a way that the potential and its first two derivatives are continuous at the cut off. We study the system in a temperature range $T \in \{0.45, 2.0\}$. The system size we chose is $N = 108000$. Simulations with 'soft pinning' particles are basically the ternary version of the same model, but $\alpha, \beta \in A, B, C$ with $\epsilon_{\alpha\beta} = \epsilon_{BC} = \epsilon_{CC} = 1.0$ and $\sigma_{AC} = 1.10$, $\sigma_{BC} = 1.04$ and $\sigma_{CC} = 1.20$ and all other parameters are same as above.

(ii) 2dmKA – This two dimensional model is similar to the **3dKA** model but the particle number ratio is 65 : 35. Such a number ratio is chosen to avoid any crystallization in the studied temperature ranges $T \in \{0.45, 2.0\}$. The system size tin this case is $N = 10000$.

(iii) 3dR10 – This model is a 50 : 50 binary mixture [2] in three dimensions where the particles interacts via

$$V_{\alpha\beta}(r) = \epsilon_{\alpha\beta}\left(\frac{\sigma_{\alpha\beta}}{r}\right)^{10}. \tag{2}$$

The parameters for this model are, $\epsilon_{\alpha\beta} = 1.0$, $\sigma_{AA} = 1.0$, $\sigma_{AB} = 1.22$ and $\sigma_{BB} = 1.40$. Here the potential is cut off at a distance $r_{cut} = 1.38\sigma_{\alpha\beta}$ using a similar quadratic polynomial so that the potential and its first two derivatives are continuous at the cutoff radius. The particles number density here is $\rho = 0.81$. The range of temperatures we study here is $T \in \{0.52, 2.0\}$. The system size for this model is $N = 1000 - 108000$. For soft pinning analysis we studied the ternary version of this model and use $\epsilon_{\alpha\beta} = 1.0$ and $\sigma_{CC} = 1.60$, $\sigma_{AC} = 1.30$ and $\sigma_{BC} = 1.50$ with all other parameters being kept same as above.

(iv) 2dR10 – This model is similar to **3dR10** model in two dimensions with number density $\rho = 0.85$. The temperature range for this model is $T \in \{0.520, 2.000\}$ and the system size is $N = 128 - 10000$. For all the model systems the studied temperature ranges fall within the moderately supercooled regime.

II. POINT-TO-SET METHOD

In the main text, we have compared the length scales obtained using the new method with the one obtained using the well-known Point-To-Set(PTS) [3] method, described below. This method was originally introduced by Bouchaud

*Electronic address: smarajit@tifrh.res.in

and Biroli [4]. The idea is first to equilibrate the system at the desired temperature. Then one needs to define a region of radius R outside which all the particles are pinned at their respective equilibrium positions. The particles inside the spherical region are then allowed to equilibrate via natural dynamics. For small values of R, the particles inside the spherical region will remain correlated due to the frozen boundary effect created by the outside particles. They will not be able to decorrelate even at large enough time. The decorrelation will only happen when the radius R of the cavity is allowed to be sufficiently large compared to the inherent length scale ξ_{PTS} of the system. The static length scale is then identified as the value of R where this crossover occurs. To systematically characterize it, one needs to calculate a static overlap correlation function defined as

$$q_c(R) = \lim_{t\to\infty} \frac{1}{ml^3\rho} \sum_{i=1}^{N} \langle n_i(0)\rangle \langle n_i(t)\rangle , \tag{3}$$

where m is the number of small boxes of linear size l in the central region of the cavity, ρ is the number density of the liquid, and $\langle ...\rangle$ denotes the averaging. $n_i(t) = 1$ if a box is occupied by a particle at time t and $n_i(t) = 0$ if it is empty. The l is chosen in such a way that one box can accommodate only one particle. The function is calculated in the limit $t \to \infty$. The function will decay from 1 to the bulk value $q_0 = \rho l^3$, the probability that a box will be occupied. For each value of R this is then fitted to an exponential function $q_c(R) - q_0 = A \exp\left[-\left(\frac{R-1.0}{\xi_{PTS}}\right)^{\eta}\right]$ to extract the length scale ξ_{PTS} (for details see [5]). Some of the length scales compared in the main text were taken from Ref. [6].

III. CONFIGURATIONAL OVERLAP

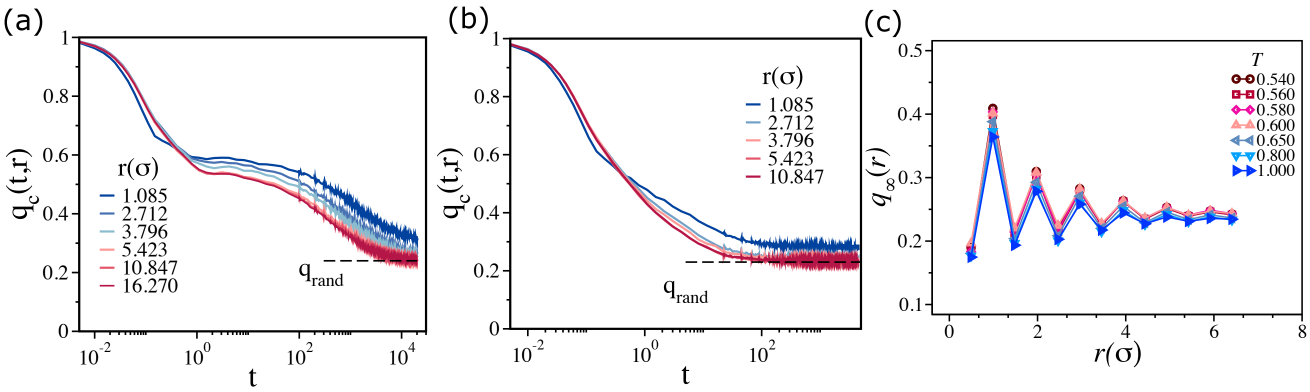

FIG. S1: (a) and (b) shows the variation of $q_c(t, r)$ as a function of t for two different temperatures $T = 0.600$ and $T = 1.000$ respectivley for the **2dR10** model. (c) shows q_∞ as a function of r from the pinning site.

Configurational overlap $q_c(t, r)$ described in main text is shown at various values of r for two different temperatures ($T = 0.600$ and $T = 1.00$) for **2dR10** model in Fig. S1 (a) and (b). For high temperatures, the function decays very fast and reaches the bulk value. Whereas, for the low temperature ($T = 0.600$), we see the basic feature of glassy dynamics – the two-step relaxation. The $q_\infty(r)$ for the same model is shown in panel (c). As mentioned in the main text, in Figure 1(c), the data are scaled along the y-axis via certain scale factors for better visibility. The scale factors are $4.653, 2.653, 1.629, 1.0, 0.711, 0.585, 0.436, 0.326, 0.326$ for $T = 0.450, 0.470, 0.500, 0.550, 0.600, 0.700, 0.800, 1.000, 1.250$ respectively.

IV. RELAXATION TIME AS A FUNCTION OF RADIAL DISTANCE (r) FROM THE PINNING SITE AND SCALING ANALYSIS

In Fig. S2 (a), we have shown the temperature dependence of relaxation time at a distance $r = 0.77\sigma$ from the pinning site with the bulk relaxation time for **3dR10** model. Indeed, for all temperatures, the relaxation time near the pinning site is larger than the bulk value. Still, the temperature dependence is very similar to the bulk relaxation time, as shown in the inset of the same figure. In Fig. S2 (b), we have demonstrated the radial dependence of average

relaxation time ($\tau_\alpha(r,T)$) computed from a pinning site for various temperatures for the **2dR10** model. The radial dependence of $\tau_\alpha(r,T)$ clearly shows that with lowering the temperature, the effect of the pinning site is felt up to a longer distance, indicating the interplay of the underlying static length scale.

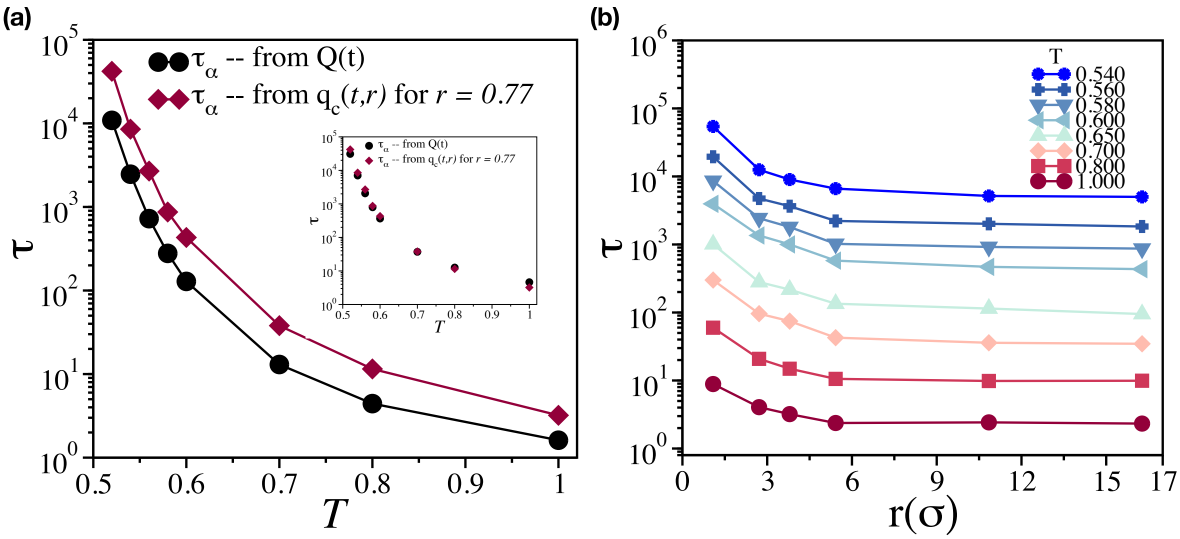

FIG. S2: (a) Comparison of the temperature dependence of the relaxation time at a certain radial distance from a pinning site with the bulk relaxation time for the **3dR10** model. Inset shows the scaled version to show that they have almost the same temperature dependence. (b) Shows the dependence of relaxation time of particles situated at a radial distance r from a pinning site for all the studied temperatures for the **2dR10** model.

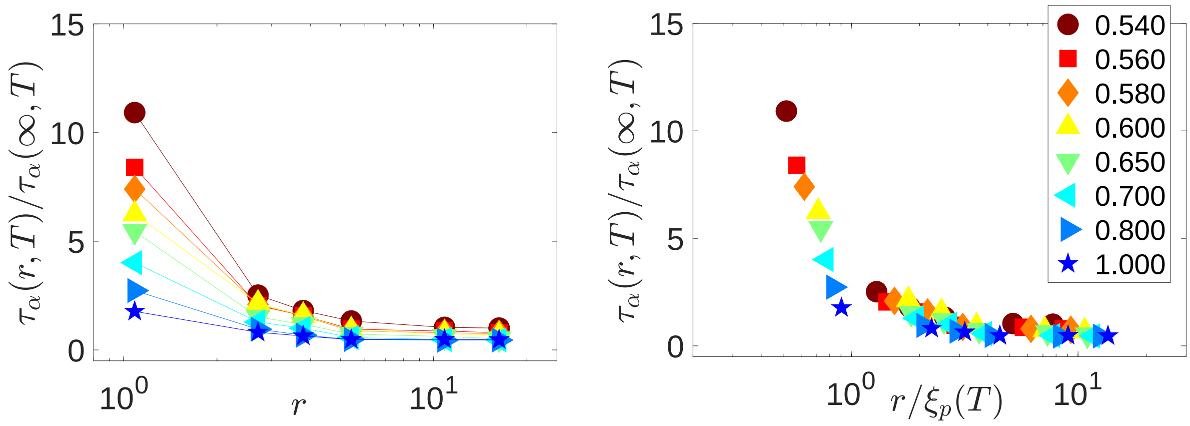

FIG. S3: (a) Shows $\frac{\tau_\alpha(r,T)}{\tau_\alpha(\infty,T)}$ as a function of r from the pinning site. Here $\tau_\alpha(\infty,T)$ is chosen to be same as the bulk value of $\tau_\alpha(T)$. (b) Shows data collapse using the same pinning length scale obtained from the decay of $|q - q_{rand}|$ as a function of r (see Fig. 1 in the main text). Note that we do not have any adjustable parameters in this data collapse. Even then, the data collapse is found to be quite good. This suggests that the spatial dependence of relaxation time is indeed governed by the underlying static length scale of the system at that particular temperature.

As discussed in the main text, if the relaxation dynamics of the system is controlled by an underlying static length scale then one can expect that the spatial dependence of relaxation time from a pinning site will obey the following scaling relation

$$\tau^p_\alpha(r,T) = \tau_\alpha(T)\mathcal{F}\left[\frac{r}{\xi_s(T)}\right], \tag{4}$$

where ξ_s is the underlying static length scale and $\mathcal{F}(x)$ is a scaling function which goes to 1 as $x \to \infty$. This assumption is used in the main text to derive the final scaling relation between the relaxation time of the whole system in the presence of pinning particles and the underlying static length scale as given by

$$\frac{\tau_\alpha(c,T)}{\tau_\alpha(T)} \simeq 1 + \kappa c \xi_s^3 \quad \text{or} \quad \log\left[\frac{\tau_\alpha(c,T)}{\tau_\alpha(T)}\right] \simeq \kappa c \xi_s^3, \tag{5}$$

where $\kappa = \rho \int_0^\infty [\mathcal{F}(x) - 1] x^2 dx$. Thus, the validity of the scaling relation scaling relation in Eqn. (5) crucially depends on the validity of Eqn. (4). If the assumption made in Eqn. (4) is correct then one might be able to do a scaling collapse of the r dependent τ_α data shown in Fig. S2 (b) using the static length scale. To check this we do the scaling analysis in 2dR10 model with random pinning. It is important to note that in the presence of random pinning the appropriate static length scale is the pinning length scale, ξ_p [6]. The data collapse using the ξ_p in 2dR10 model is shown in the Fig. S3 (a) and Fig. S3 (b). The data collapse obtained in Fig. S3 (b) is reasonably good without any adjustable parameters suggesting the validity of the scaling assumption, Eqn. (4) and the robustness of the scaling relation Eqn. (5). One can see that collapse is bad for $T = 0.8$ and 1.0, which are high temperatures, and measurement of spatial dependence of relaxation time at these high temperatures can be noisy as correlation length will be very small.

V. EXPERIMENTAL SETUP AND DATA COLLECTION:

The schematic of the experimental setup used in this work is shown in Fig. S4. The dielectric constant and loss are measured using the parallel plate capacitor kept inside Aluminium sample holder as shown in Fig.S4. The electrodes of the capacitor are made of stainless steel to avoid any kind of chemical reaction with the samples. Teflon plates are used to avoid contact between electrodes and the sample holder. The separation between two electrodes is $1mm$, and the area of the electrodes is $36cm^2$. The sample holder was connected to a solid massive Aluminium rod of very large heat capacity. This whole structure is kept inside a Dewar flask to keep it thermally isolated from the environment.

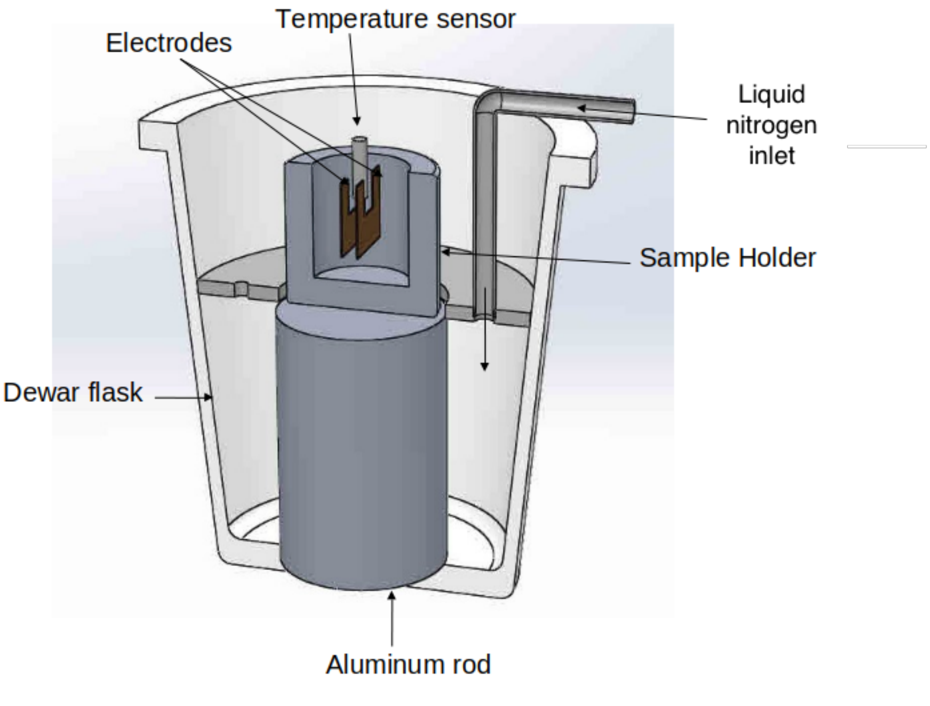

FIG. S4: Schematic presentation of the home-built experimental setup containing a dewar flask which contains liquid nitrogen during experimental runs. An aluminium rod connected to the sample chamber is used for sample chamber cooling. A two-electrode plate connected to a potentiostat (Biologic SP300) is used for dielectric spectroscopy at different sample temperatures.

The sample was poured inside the sample holder, which contains the parallel plate capacitor. After that, the heavy aluminium rod was cooled using liquid nitrogen. As the aluminium cup and the sample inside it were connected, it

cooled the samples automatically. The temperature of the sample was monitored using an electronic temperature detector. Once the temperature reached the desired temperature, we stopped cooling. After that, the temperature starts to increase due to the heat transfer to the environment. As the whole system was suitably thermally insulated, the rate of this increment was low, which allowed us to have enough time to collect the data. Using this method at each temperature window, we were able to maintain the temperature for around 3-4 minutes to perform our measurements. We consider dielectric storage modulus (ϵ') as the suitable quantity to measure the proposed pinning susceptibility [7, 8]. We stress that as $\epsilon'(t)$ and $q(t)$ are both two point correlation functions, one can thus compute the pinning susceptibility from $\epsilon'(t)$ as well. The susceptibility is then defined as

$$\chi_p^{Expt}(T,t) = \left(\frac{\partial \epsilon'(T,c,t)}{\partial c}\right)\Big|_{c=0} = \frac{\epsilon'(c,T,t) - \epsilon'(c=0,T,t)}{c}\Big|_{c=0}, \tag{6}$$

where c is the concentration of Sorbitol. We measured ϵ' of Glycerol-Sorbitol mixture for a range of temperature $T \in \{203K...248K\}$. We varied Sorbitol concentration (c) from 0 to 15%. The range of ϵ' is re-scaled within $0-1$ using a modified form of Havriliak-Negami fitting function for the dielectric relaxation[9] (see Sec.VII for details). Note that the fitting procedure is used only to obtain the low and high frequency limit of the dielectric constant for the normalization purpose. No fitting is involved any other part of the experimental data analysis.

VI. EXTRACTION OF EXISTING EXPERIMENTAL DATA FROM LITERATURE

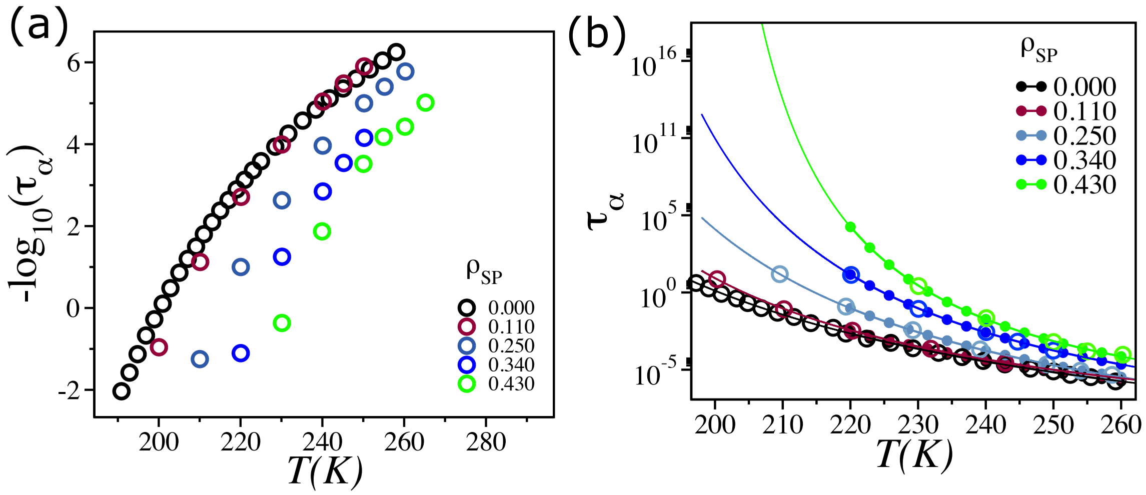

FIG. S5: (a) Raw data extracted from Fig.2 in Ref. [10] for Glycerol-Sorbitol mixture. (b) Shows the method to extract τ_α at desired temperatures using VFT fitting of the raw data.

We use our scaling relation to extract length scales for numerical and experimental systems in the main text. We studied three different experimental systems –(i) Glycerol-Sorbitol mixture, (ii) Butnaol-Hexanol mixture, and (iii) Glycerol-Glucose mixture. We performed an experiment in our lab for the Glycerol-Sorbitol mixture and Glycerol-Glucose mixture. We also analysed data extracted from previously published experimental data. We use WebPlotDigitizer software to extract raw data from the published paper. The Glycerol-Sorbitol data was extracted from Ref. [10]. We first extracted the raw data from Fig. 2 of Ref.[10]. We then fitted the T vs. τ_α data to the VFT equation given by

$$\tau_\alpha(T) = \tau_0 \exp\left(\frac{AT}{T - T_{VFT}}\right). \tag{7}$$

The VFT fitted data was used to extract time-scales (τ_α) at desired temperatures for all the studied fraction ρ_{SP} Sorbitol in the Glycerol. We show the complete procedure in Fig. S5 (a) and (b). Fig. S5 (a) shows the raw data. In Fig. S5 (b), we show the VFT fit of the data to Eqn. (7). The open circle represents the raw data, and the

solid lines are the fit to the Eqn. (7). The small filled circle represents the extracted data at desired temperatures. We follow the same method for extracting the Butanol and Hexanol mixture shown in the main text from Ref. [11].

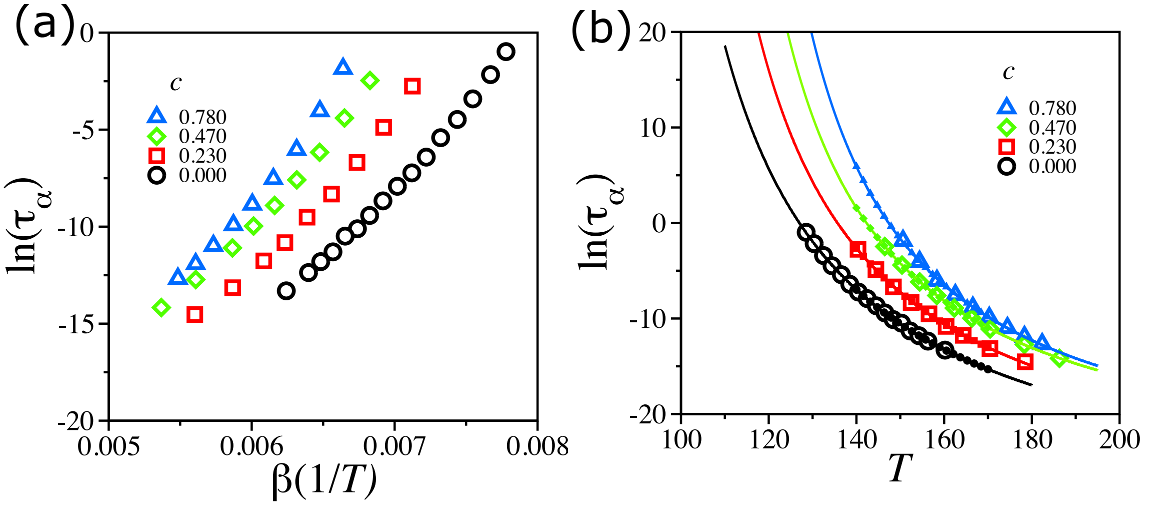

FIG. S6: (a) Raw data extracted from Fig. 3 (a) in Ref. [11] for Butanol-Hexanol mixture. (b) Shows methods to extract τ_α at desired temperatures using VFT fitting of the raw data.

The complete extraction methods are shown in Fig. S6. Note that for this mixture, we do not have data for small concentrations of Hexanol in Butanol. Nevertheless, we used this data to see if the dependence of the relaxation time of the concentrations of Hexanol in Butanol can be rationalized using the same argument that is employed to understand the data of the Glycerol-Sorbitol mixture. The results suggest that the scaling arguments hold even for this data set, leading to the extraction of the underlying static length scale. We could not independently check the validity of this obtained length scale, and future studies, particularly at a smaller concentration of Hexanol in Butanol, will be needed.

VII. DIELECTRIC LOSS AND DIELECTRIC CONSTANT OF GLYCEROL+SORBITOL MIXTURE

In this section, we have given all the data of dielectric constant and dielectric loss (ϵ' and ϵ'') for the pure Glycerol and the Glycerol-Sorbitol mixture with varying concentrations of Sorbitol. Note that the data of dielectric constant (ϵ') is normalized following a modified version of Havriliak-Negami fitting formula [9] as given below

$$\epsilon'(\omega) = \epsilon_\infty + \frac{\epsilon_s - \epsilon_\infty}{1 + (\omega\tau)^\alpha}, \tag{8}$$

where ϵ_∞ and ϵ_s are the high and low-frequency limits of ϵ'. τ is the typical relaxation time and α is a fitting exponent. This form is simplified, and we highlight that it fits the data quite well. Note that this fitting is used only to get ϵ_∞ and ϵ_s for normalizing the data to the range $[0, 1]$. This normalization is needed to compute the pinning susceptibility $\chi_p \equiv \frac{\partial \epsilon'}{\partial c}$, where c is the concentration of the co-solvent (Sorbitol in Glycerol-Sorbitol mixture and Hexanol in Butanol-Hexanol mixture). Any other data analysis does not involve the results obtained from this fitting procedure. In the left panels of Figs. S7 and S8, we have plotted $\epsilon'_n = \frac{\epsilon - \epsilon_\infty}{\epsilon_s - \epsilon_\infty}$ for various concentrations of Sorbitol in supercooled Glycerol. The high frequency value of the dielectric constants ϵ_∞ as a function of temperature T is shown in Fig. S9.

We also highlight that one can obtain the relaxation time of the liquid mixture from this fitting procedure, but we have rather used the peak value of ϵ'' to determine the structural relaxation time, τ_α just to avoid any error that might come due to fitting procedure. Also, note that to get a very precise value of the peak position in ϵ'', we have fitted to the peak of the dielectric loss using a quadratic function and then determined the maximum position and the corresponding time (frequency) where the peak appears. This also removes any human intervention while estimating the relaxation time, τ_α of the Glycerol-Sorbitol mixture from the peak position of the dielectric loss. In the right panels of Figs. S7 and S8, we have plotted ϵ'' for all the studied concentrations of Sorbitol in Glycerol.

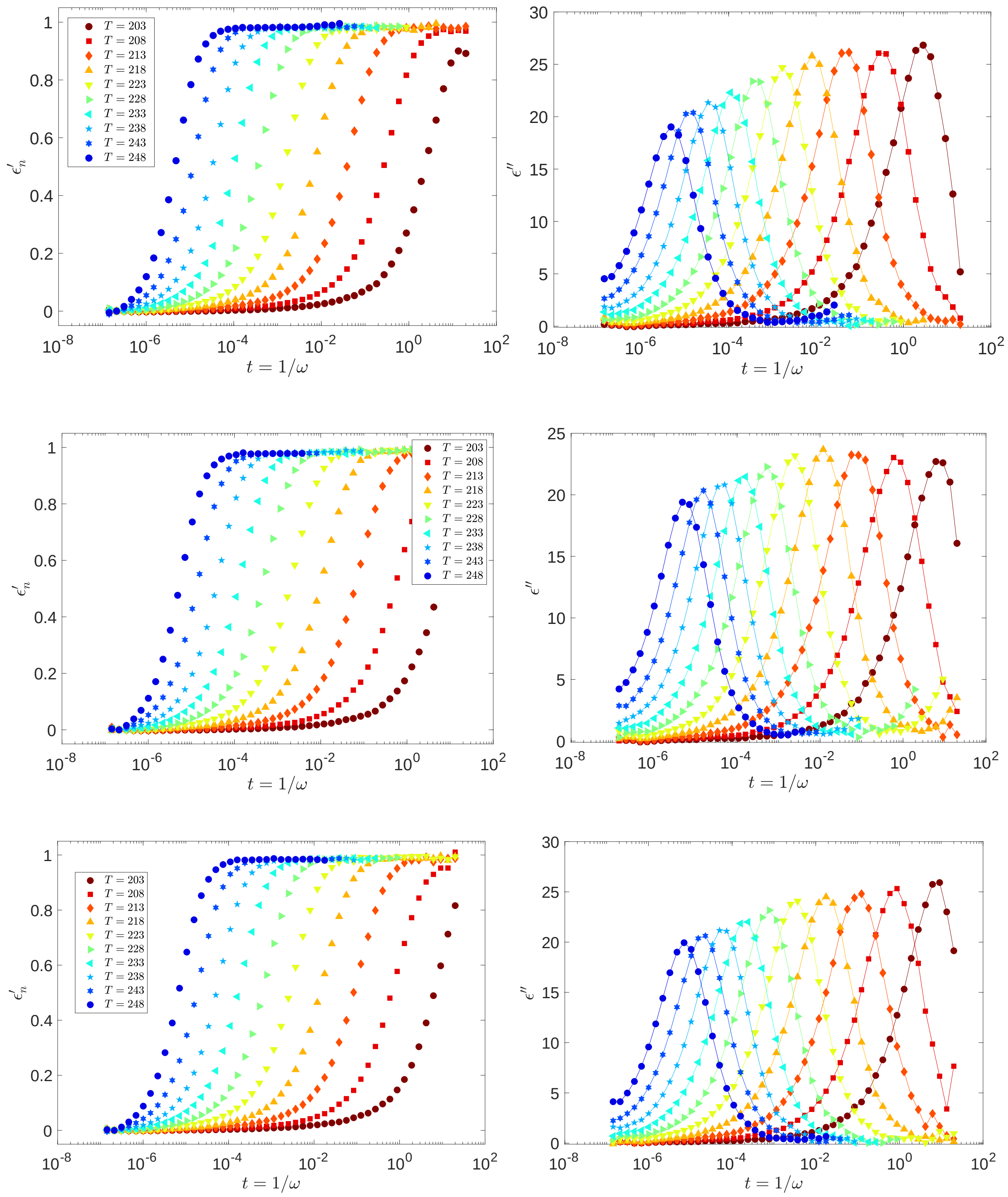

FIG. S7: Top panels: Normalized dielectric constant (ϵ') and dielectric loss (ϵ'') for pure Glycerol sample. Middle panels: Similar data for Glycerol + 2% Sorbitol mixture. Bottom panels: For Glycerol + 5% Sorbitol mixture.

VIII. PINNING SUSCEPTIBILITY FOR VARIOUS CONCENTRATIONS OF SORBITOL

In Fig. S10 we have shown the pinning susceptibility, $\chi_p \equiv \frac{\partial \epsilon'_n}{\partial c}$ with c being the concentration of Sorbitol in supercooled Glycerol. We have shown the plots of pinning susceptibility for $c = 7.5\%, 10\%$ and 15% ($c = 5\%$ is

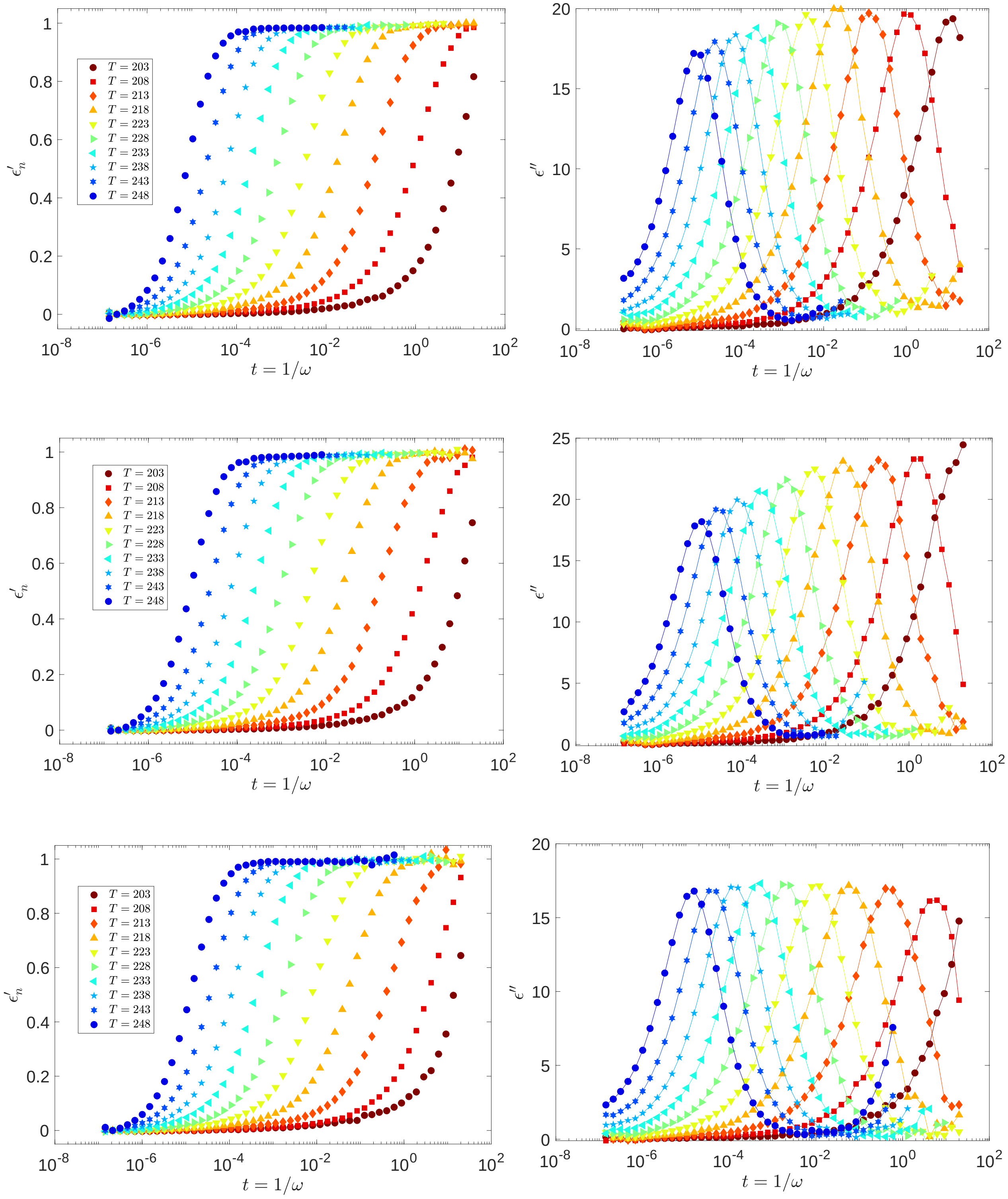

FIG. S8: Top panels: Normalized dielectric constant (ϵ') and dielectric loss (ϵ'') for Glycerol + 7.5% Sorbitol mixture sample. Middle panels: Similar data for Glycerol + 10% Sorbitol mixture. Bottom panels: For Glycerol + 15% Sorbitol mixture.

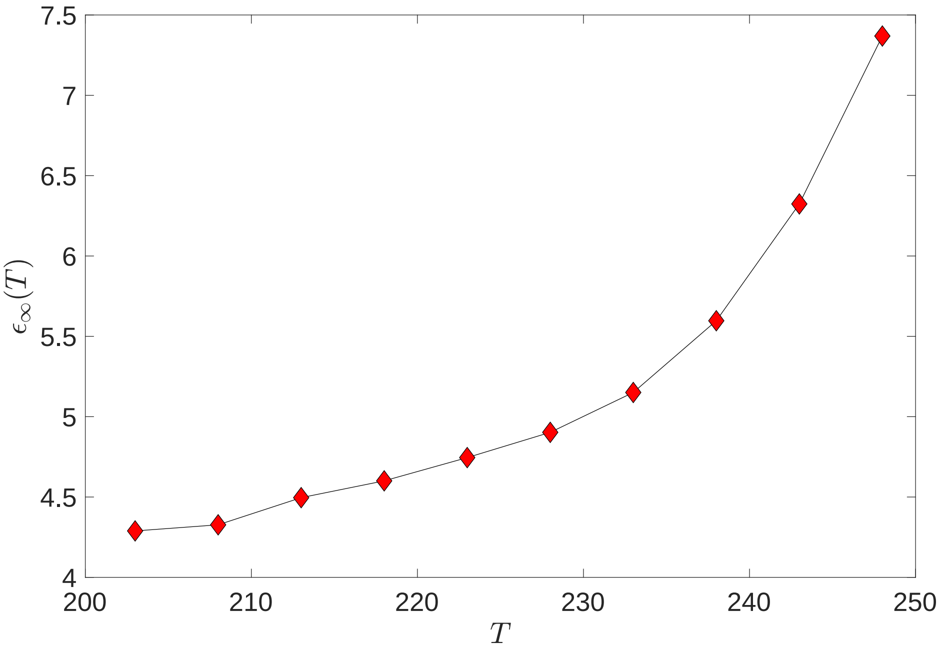

FIG. S9: High frequency value of the dielectric constants ϵ_∞ as a function of temperature T

given in the main article) as pinning susceptibility for smaller concentration ($c < 5\%$) of Sorbitol is noisy for reliable estimation of it.

IX. CONSISTENCY CHECK

To check whether our experimental data is consistent with the previously published data, we first compare the relaxation time τ_α for pure Glycerol ($c = 0.00$) and one fraction of the Glycerol-Sorbitol mixture ($c = 0.100$(our) and 0.110(extracted)). Apart from an overall scaling factor, our data matches well with the previously published experimental data. In Fig. S11 we show the comparison.

We then compare other important quantities such as glass transition temperature T_g, the VFT divergence temperature T_{VFT}, and the kinetic fragility K_{VFT} for our experimental data and the published data. The glass transition temperature T_g is defined as $\tau_\alpha(T_g) = 100s$. T_{VFT} is extracted as a fitting parameter using Eqn. (7) to fit T vs. τ_α data (See Fig. S5). The kinetic fragility defined as $K_{VFT} = \frac{1}{A}$ in the Eqn. (7) also obtained as a fitting parameter. In Fig. S12, we show such a plot for the comparison of various quantities. Note that apart from an overall scaling factor, the T_g values match nicely with the reported data. The divergence temperature T_{VFT} (no scaling factor) and the kinetic fragility K_{VFT} also match nicely.

X. SCALING COLLAPSE

We have reshown the data collapse of relaxation time of Glycerol with increasing concentrations of Sorbitol and performed the data collapse including the lowest temperature data ($T = 203K$). As we do not have complete data for this temperature, we did not show this temperature point in the main manuscript but shown here. The data collapse remain as good as before although the lowest temperature data points are a bit noisy. The corresponding correlation lengths are shown in the right panel. The correlation length systematically grow with lowering the temperature.

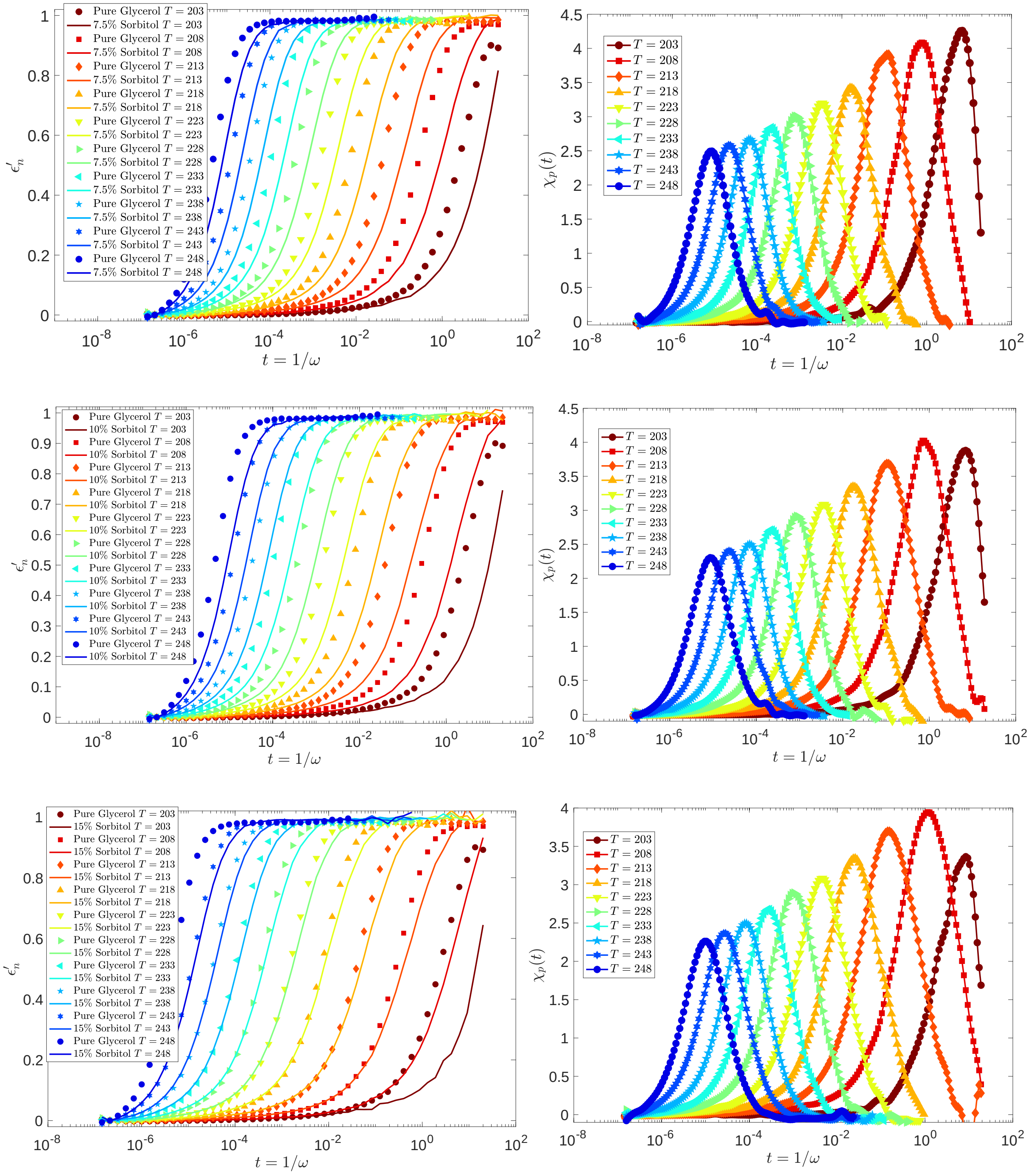

FIG. S10: Left panels: Normalized dielectric constant for pure Glycerol (symbols) and Glycerol-Sorbitol mixture (line) are shown for various concentrations of Sorbitol $c = 7.5\%$ (top panel), 10% (middle panel) and 15% (bottom panel) and the corresponding Pinning Susceptibility, $\chi_p(t)$ for these Sorbitol concentrations are shown in the respective right panels.

XI. EFFECT OF INTER-ATOMIC INTERACTION ON PINNING SUSCEPTIBILITY

As discussed in the main text, the inter-atomic interaction between the soft pinning particles and the host liquid medium could possibly have a significant effect on the pinning susceptibility. If it is true, then our estimation of correlation lengths would also be affected depending on the inter-atomic interaction strength. To check the possible

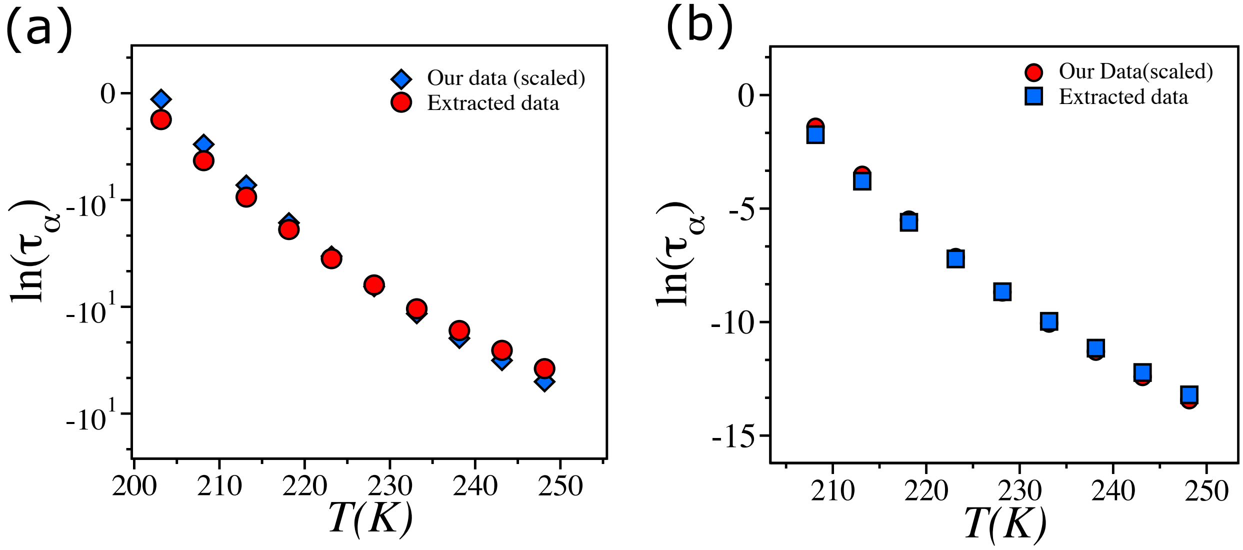

FIG. S11: Comparison of τ_α for pure Glycerol (a) and for $c = 0.100$ of Glycerol-Sorbitol mixture (b).

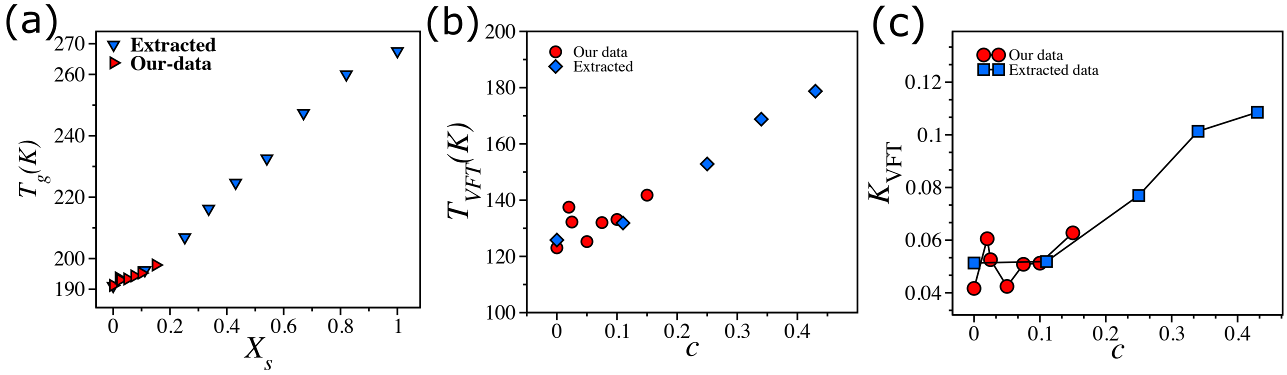

FIG. S12: Comparison of T_g (scaled) (a), T_{VFT} (b) and kinetic fragility K_{VFT} (c) between our data and the previously published data.

effect, we have performed another set of simulations by changing the interaction strength between soft pinning particles and the host medium (σ_{AC} and σ_{BC}) for the **3dKA** ternary model. All other parameters are kept as before (see the Method section). In left panel of Fig. S14 we show $\chi_p(t)$ as a function of t at various T for **3dKA** model with $\sigma_{AC} = \sigma_{BC} = 1.4$. The growth of $\chi_p(t)$ with lowering temperatures suggests the growth of the length scale. Moreover, the behavior does not change as we change the interatomic interaction strength. The corresponding comparison of length scales is shown in the right panel of Fig. S14. Note that the length scales match nicely in the entire temperature ranges. We also have verified the effect of interatomic interactions on the pinning susceptibility for the experimental system (see main text).

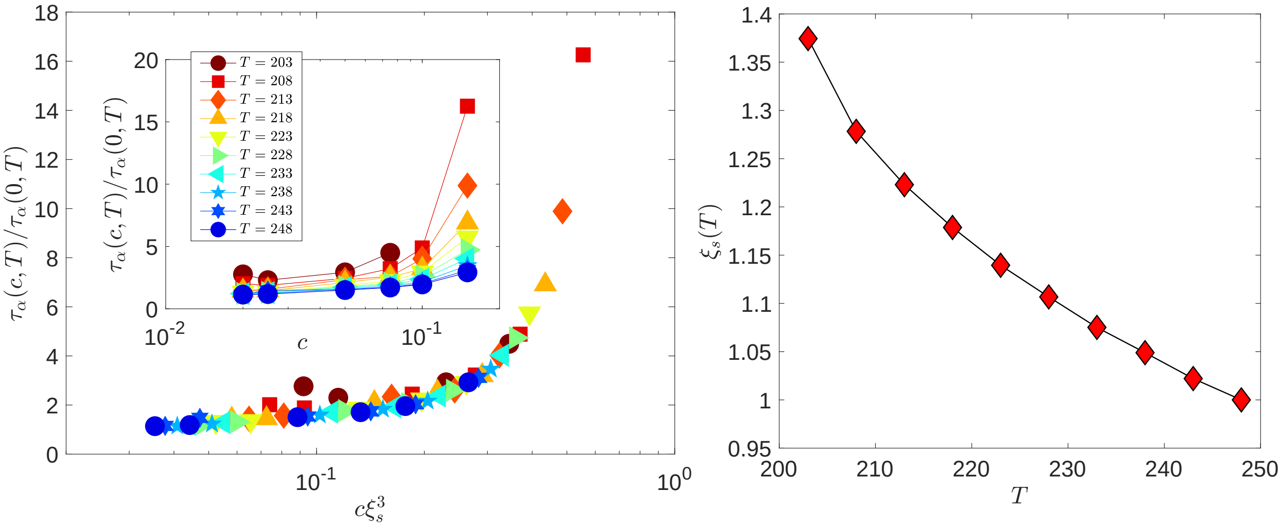

FIG. S13: **Data Collapse** Left panel: τ_α scaling collapse shown for our data after including the lowest temperature ($T = 203K$) data points which are incomplete as we could not get the largest concentrations data for this temperature. Right panel: The growth of the correlation length.

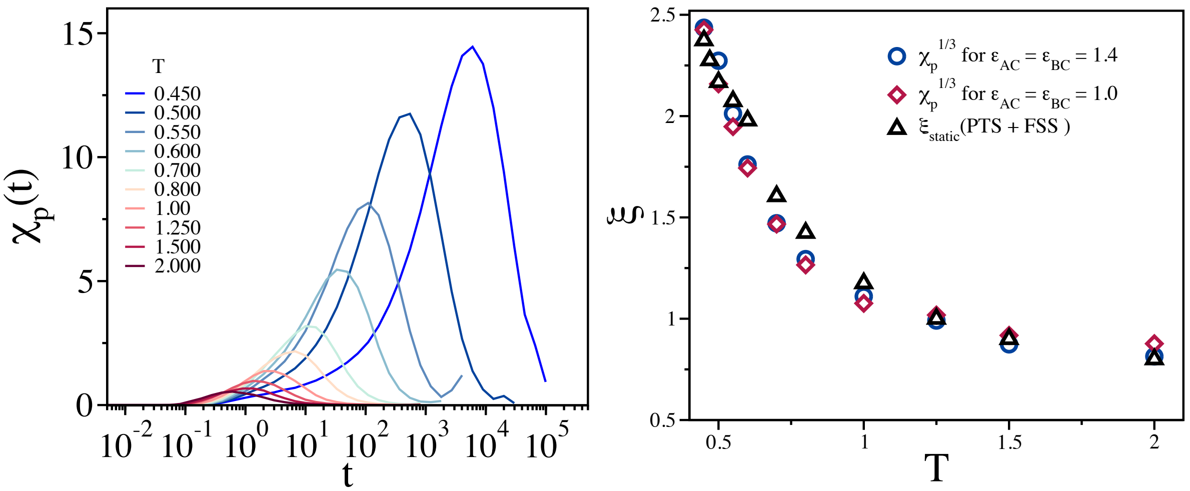

FIG. S14: **Investigating the effect of interatomic interaction between soft pinning particles and the host medium.** The pinning susceptibility $\chi_p(t)$ as a function of t with $\sigma_{AC} = \sigma_{BC} = 1.4$ for **3dKA** ternary model (left panel). Comparison of length scales ξ from $(\chi_p^{max})^{1/3}$ for two different values of σ_{AC} and σ_{BC} with the length scales obtained via PTS and FSS (right panel). The length scale does not change for the different values of interatomic interactions.

[1] W. Kob and H. C. Andersen, Phys. Rev. E **51**, 4626 (1995), URL https://link.aps.org/doi/10.1103/PhysRevE.51.4626.

[2] S. Karmakar, E. Lerner, and I. Procaccia, Physica A: Statistical Mechanics and its Applications **391**, 1001 (2012), ISSN 0378-4371, URL http://www.sciencedirect.com/science/article/pii/S0378437111008557.

[3] G. Biroli, J.-P. Bouchaud, A. Cavagna, T. S. Grigera, and P. Verrocchio, Nature Physics **4**, 771 (2008), ISSN 1745-2481, URL https://doi.org/10.1038/nphys1050.

[4] J.-P. Bouchaud and G. Biroli, The Journal of Chemical Physics **121**, 7347 (2004), https://doi.org/10.1063/1.1796231, URL https://doi.org/10.1063/1.1796231.

[5] G. Biroli, S. Karmakar, and I. Procaccia, Phys. Rev. Lett. **111**, 165701 (2013), URL https://link.aps.org/doi/10.1103/PhysRevLett.111.165701.

[6] S. Chakrabarty, R. Das, S. Karmakar, and C. Dasgupta, The Journal of Chemical Physics **145**, 034507 (2016), https://doi.org/10.1063/1.4958632, URL https://doi.org/10.1063/1.4958632.

[7] L. Berthier, G. Biroli, J.-P. Bouchaud, L. Cipelletti, D. E. Masri, D. L'Hôte, F. Ladieu, and M. Pierno, Science **310**, 1797 (2005), ISSN 0036-8075, https://science.sciencemag.org/content/310/5755/1797.full.pdf, URL https://science.sciencemag.org/content/310/5755/1797.

[8] S. Albert, T. Bauer, M. Michl, G. Biroli, J.-P. Bouchaud, A. Loidl, P. Lunkenheimer, R. Tourbot, C. Wiertel-Gasquet, and F. Ladieu, Science **352**, 1308 (2016), ISSN 0036-8075, https://science.sciencemag.org/content/352/6291/1308.full.pdf, URL https://science.sciencemag.org/content/352/6291/1308.

[9] S. Havriliak and S. Negami, Polymer **8**, 161 (1967), ISSN 0032-3861, URL http://www.sciencedirect.com/science/article/pii/0032386167900213.

[10] K. Duvvuri and R. Richert, The Journal of Physical Chemistry B **108**, 10451 (2004), ISSN 1520-6106, URL https://doi.org/10.1021/jp031366b.

[11] S. Katira, J. P. Garrahan, and K. K. Mandadapu, Phys. Rev. Lett. **123**, 100602 (2019), URL https://link.aps.org/doi/10.1103/PhysRevLett.123.100602.
